# MicroRNA319-regulated TCPs interact with FBHs and PFT1 to activate *CO* transcription and control flowering time in *Arabidopsis*

**DOI:** 10.1371/journal.pgen.1006833

**Published:** 2017-05-30

**Authors:** Jie Liu, Xiliu Cheng, Pan Liu, Dayong Li, Tao Chen, Xiaofeng Gu, Jiaqiang Sun

**Affiliations:** 1 National Key Facility for Crop Gene Resources and Genetic Improvement, Institute of Crop Science, Chinese Academy of Agricultural Sciences, Beijing, China; 2 Biotechnology Research Institute, Chinese Academy of Agricultural Sciences, Beijing, China; 3 State Key Laboratory of Plant Genome, Institute of Genetics and Developmental Biology, Chinese Academy of Sciences, Beijing, China; Universität Potsdam, GERMANY

## Abstract

The transcription factor CONSTANS (CO) is a central component that promotes *Arabidopsis* flowering under long-day conditions (LDs). Here, we show that the microRNA319-regulated TEOSINTE BRANCHED/CYCLOIDEA/PCF (TCP) transcription factors promote photoperiodic flowering through binding to the *CO* promoter and activating its transcription. Meanwhile, these TCPs directly interact with the flowering activators FLOWERING BHLH (FBHs), but not the flowering repressors CYCLING DOF FACTORs (CDFs), to additively activate *CO* expression. Furthermore, both the TCPs and FBHs physically interact with the flowering time regulator PHYTOCHROME AND FLOWERING TIME 1 (PFT1) to facilitate *CO* transcription. Our findings provide evidence that a set of transcriptional activators act directly and additively at the *CO* promoter to promote *CO* transcription, and establish a molecular mechanism underlying the regulation of photoperiodic flowering time in *Arabidopsis*.

## Author summary

Plants monitor day-length changes (photoperiod) throughout the year to precisely align their flowering time, which is crucial for successful reproduction. In *Arabidopsis*, some components, such as CONSTANS (CO), have been proved to play central roles in promoting the photoperiodic flowering under long-day conditions (LDs). In this study, we demonstrate that the microRNA319-regulated TEOSINTE BRANCHED/CYCLOIDEA/PCF (TCP) transcription factors directly bind to the *CO* promoter. Meanwhile, these TCPs physically interact with the flowering activators FLOWERING BHLH (FBHs) and the flowering regulator PHYTOCHROME AND FLOWERING TIME 1 (PFT1) to form a complex to activate *CO* transcription and promote photoperiodic flowering under LDs. Our results emphasize the importance of miR319-regulated TCPs in regulating plant flowering time.

## Introduction

Flowering is a transition from the vegetative to the reproductive phase in the plant life cycle, which is crucial for successful reproduction. Genetic approaches in the model plant *Arabidopsis*, in which flowering is often promoted under long-day (LD) but is delayed during short-day (SD) conditions, reveal that CONSTANS (CO) plays crucial roles in photoperiod monitoring and flowering time determination [1–3].

In *Arabidopsis*, *CO* encodes a B-box-type zinc finger transcriptional activator [4]. The *co* mutant lines flower late under LDs, whereas the plants overexpressing *CO* display early flowering phenotype in both LDs and SDs [4, 5]. Under LDs, *CO* displays a biphasic diurnal expression pattern that its transcript levels first rise at the late afternoon to form a small peak in the light period, and a second peak appears during the midnight [5]. Several studies have revealed that the CO protein stabilization is tightly controlled in a light-dependent manner by a number of factors, such as phytochrome A (PHYA), cryptochrome 2 (CRY2) and FKF1 (FLAVIN-BINDING, KELCHREPEAT, F-BOX1) and CONSTITUTIVE PHOTOMORPHOGENIC 1 (COP1) [6–10]. Therefore, the induction of *CO* mRNA levels at dusk under LDs but not the peak expression at night is essential for the CO protein accumulation and subsequent photoperiodic flowering promotion.

To date, several components have been identified to precisely regulate the diurnal transcription of *CO* in *Arabidopsis*. The transcription factors CYCLING DOF FACTORs (CDF1-5) are the well characterized repressors of *CO* transcription [11, 12]. However, as the repressors, CDFs could not fully explain the remarkable up-regulation of *CO* transcript levels at dusk. The four basic helix-loop-helix-type (bHLH) transcription factors FLOWERING BHLH 1 (FBH1), FBH2, FBH3, and FBH4 have been identified as the *CO* transcriptional activators that preferentially bind to the E-box *cis*-elements of the *CO* promoter in the afternoon to induce the expression of *CO* [13], proposing a complicated temporal interplay among repressors and activators in restricting the *CO* transcription. However, unlike CDFs, FBHs do not show robust daily oscillation at either mRNA or protein levels, implying that their time-dependent binding preference on *CO* promoter is potentially affected by some other unidentified regulators or co-activators [13].

In addition to the transcription factors, *PHYTOCHROME AND FLOWERING TIME 1* (*PFT1*), encoding the Mediator complex subunit 25 (MED25) in *Arabidopsis*, was reported to genetically act upstream of *CO* and promote flowering [14, 15]. However, the molecular mechanisms about how PFT1 relies on the information from light signals to control flowering time through affecting *CO* transcript levels remain obscure.

The plant-specific TEOSINTE BRANCHED1/CYCLOIDEA/PCF (TCP) family transcription factors contain a conserved non-canonical bHLH domain, which mediates DNA binding or interactions with other proteins [16]. In *Arabidopsis*, the *jaw-D* mutants, in which microRNA319 (miR319) is over accumulated and five class II *TCP* genes including *TCP2*, *TCP3*, *TCP4*, *TCP10*, and *TCP24* are down-regulated, show delayed flowering phenotype [17–19]. However, the functional mode and action mechanism of these TCPs transcription factors in regulation of *Arabidopsis* flowering time remain unclear. In this study, we demonstrate that the miR319-regulated TCPs function as direct transcriptional activators of the photoperiodic flowering regulator CO to promote *Arabidopsis* flowering under the inductive photoperiod. Furthermore, these TCPs transcription factors physically interact with the flowering activators FBHs. Meanwhile, we found that these TCPs and FBHs transcription factors directly interact with the flowering time regulator PFT1 to facilitate *CO* transcription, and this conclusion is further supported by the observation that PFT1 proteins are exclusively enriched in the TCP- and FBH-binding regions of *CO* promoter under LDs. Thus, we uncover a transcriptional activation complex for direct activation of *CO* transcription to promote *Arabidopsis* photoperiodic flowering.

## Results

### The miR319-regulated TCPs promote *Arabidopsis* photoperiodic flowering

**Fig 1:**
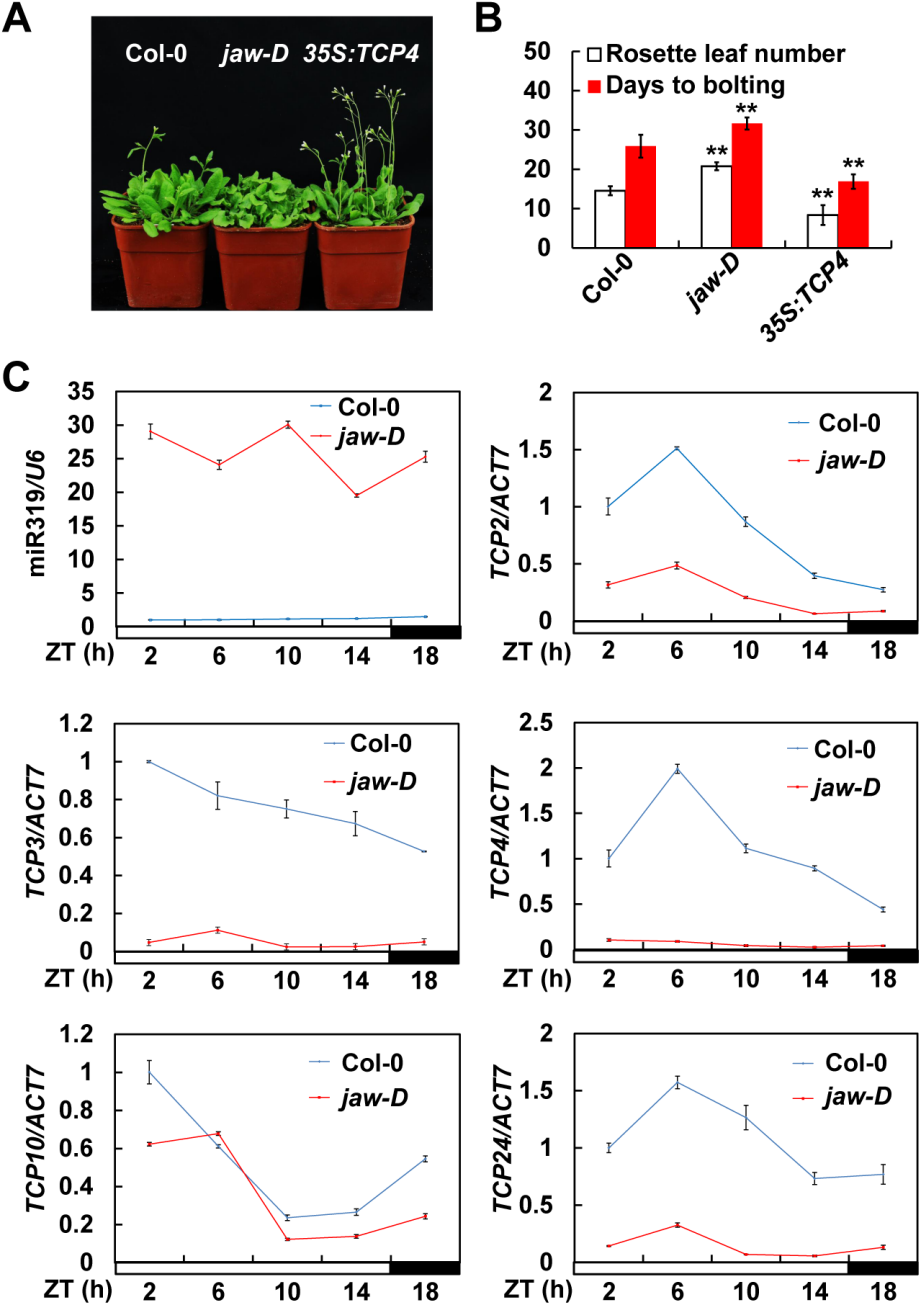
The miR319-regulated TCPs promote *Arabidopsis* photoperiodic flowering. (A) Flowering times of 30-d-old wild type (WT) Columbia-0 (Col-0), *jaw-D* and *TCP4* overexpression line (*35S*:*TCP4*) under long-day conditions (LDs). (B) Quantification of the flowering times of Col-0, *jaw-D* and *35S*:*TCP4*. The rosette leaf numbers (mean ± SD, n ≥ 40 plants) as well as the days to bolting (mean ± SD, n ≥ 15 plants) were separately calculated per genotype. Asterisks above the bars denote significant differences compared with the WT Col-0 plants at *P* < 0.01 (**, Student’s *t* test). (C) Expression levels of miR319, *TCP2*, *TCP3*, *TCP4*, *TCP10* and *TCP24* in Col-0 and *jaw-D* under LDs. The mean values in WT Col-0 at *Zeitgeber* time (ZT) 0 were set to 1 (mean ± SD, n = 3).

Previous studies have shown that the *Arabidopsis tcp4* and *jaw-D* mutants displayed delayed flowering under LDs [17–19]. To further evaluate the potential role of the miR319-regulated TCPs transcription factors in the photoperiodic flowering pathway, we examined the flowering time phenotype of *jaw-D* mutant lines under both LD (16 h light/8 h dark) and SD (8 h light/16 h dark) conditions. As expected, the *jaw-D* plants displayed an obvious late-flowering phenotype compared with wild type (WT) Columbia-0 (Col-0) under inductive LDs (Fig 1A and 1B), but flowered normally under the non-inductive SDs (S1 Fig). Next, we analyzed the expression patterns of miR319 together with the five miR319-regulated *TCPs*, including *TCP2*, *TCP3*, *TCP4*, *TCP10* and *TCP24*, in both wild type and *jaw-D* mutant plants. In consistent with previous reports [17, 19], we also showed that the five miR319-regulated *TCPs* were all significantly down-regulated in *jaw-D* plants compared with WT, coupling with the elevated miR319 levels (Fig 1C). Intriguingly, the mRNA levels of *TCP2*, *TCP4* and *TCP24* exhibited similar diurnal expression patterns (Fig 1C). Accordingly, we assumed that miR319-regulated TCPs may be involved in the *Arabidopsis* photoperiodic flowering pathway. To further confirm this hypothesis, we generated transgenic plants carrying a *35S*:*TCP4* construct [17] (S2 Fig), and observed a significantly early flowering phenotype in the *35S*:*TCP4* transgenic plants under both LDs and SDs (Fig 1A and 1B; S1 Fig). In summary, we concluded that the miR319-regulated TCPs are positive regulators of *Arabidopsis* photoperiodic flowering.

### The miR319-regulated TCPs positively regulate *CO* transcription

**Fig 2:**
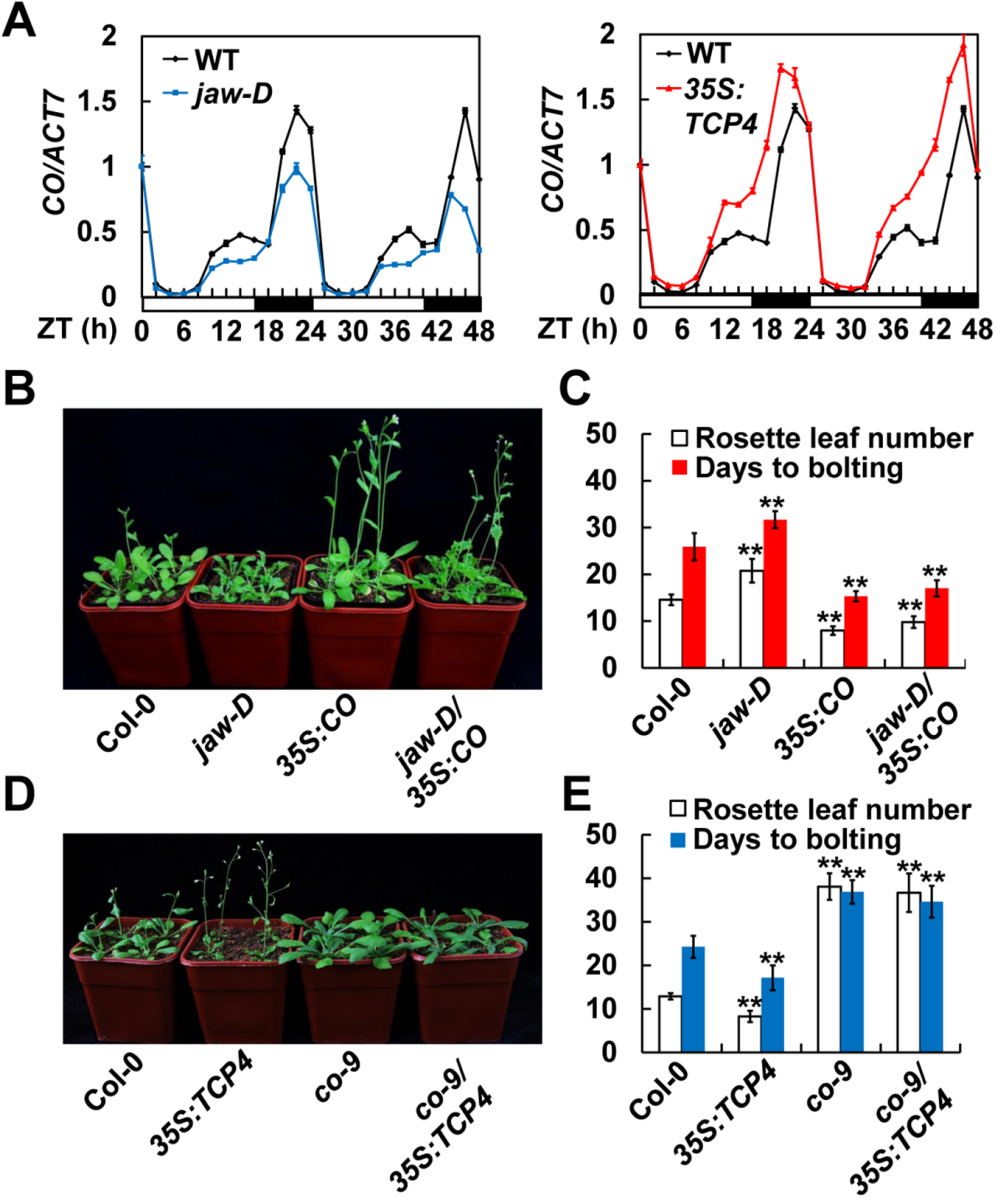
The miR319-Regulated *TCPs* act genetically upstream of CO in controlling flowering time. (A) Temporal expression patterns of *CO* in Col-0, *jaw-D*, and *35S*:*TCP4* transgenic line under LDs. (B-E) Genetic interaction of miR319-regulated TCPs and CO. The flowering phenotypes of seedlings grown under LDs were shown in (B) and (D), and the numbers of rosette leaves (mean ± SD, n ≥ 40) as well as the days to bolting (mean ± SD, n ≥ 15) were calculated in (C) and (E).

The delayed flowering phenotype of *jaw-D* plants under LDs led us to examine whether the *CO* expression is altered in the *jaw-D* mutant line. Expectedly, the transcript levels of *CO* were notably reduced in the *jaw-D* line during the time periods of *CO* mRNA peaks [*Zeitgeber* time (ZT) 12–16 and ZT 20–24], as compared with Col-0 seedlings (Fig 2A, left panel). On the contrary, the *CO* mRNA levels in the *35S*:*TCP4* line were obviously increased compared with those in WT (Fig 2A, right panel). Notably, we observed a significant up-regulation of *CO* in the *35S*:*TCP4* seedlings at dusk during the light phase (ZT 12–16) (Fig 2A, right panel). As is well known, CO directly activates the expression of its downstream targeting flowering-time gene *FLOWERING LOCUS T* (*FT*). Thus, we assumed that the *FT* transcription might be also affected in the *jaw-D* and *35S*:*TCP4* plants. As expected, *FT* expression was partially compromised in *jaw-D* mutant, but significantly up-regulated in the *35S*:*TCP4* transgenic line at different time points (S3 Fig), consistent with the altered *CO* levels in these lines. Together, these results imply that the miR319-regulated TCPs might play positive regulatory roles in activating *CO* transcription.

### The miR319-regulated TCPs act genetically upstream of CO in regulating flowering

To investigate the functional relationship between TCPs and CO *in vivo*, we tested for genetic interactions between these genes. We crossed the *CO*-overexpressing transgenic line *35S*:*CO* into the *jaw-D* background to generate the *jaw-D/35S*:*CO* plant and examined its flowering time phenotype in LDs. As expected, the *35S*:*CO* and *jaw-D* plants displayed early-flowering and late-flowering phenotypes, respectively (Fig 2B and 2C). However, all of the *jaw-D/35S*:*CO* seedlings flowered early resembling the flowering time phenotype of *35S*:*CO* plants (Fig 2B and 2C), indicating that overexpression of *CO* can rescue the late-flowering phenotype of *jaw-D*. Meanwhile, we introduced *35S*:*TCP4* into the *co-9* mutant background [20] to generate the *co-9/35S*:*TCP4* line. The flowering time analysis in LDs revealed that, similar to *co-9*, all the *co-9/35S*:*TCP4* seedlings exhibited late-flowering phenotype compared to Col-0 (Fig 2D and 2E), suggesting that the early flowering phenotype caused by *35S*:*TCP4* is largely dependent on the function of CO. Together, these data suggest that the miR319-regulated TCPs may function genetically upstream of CO to promote photoperiodic flowering.

### TCP4 directly binds to the *CO* promoter and activate *CO* transcription

**Fig 3:**
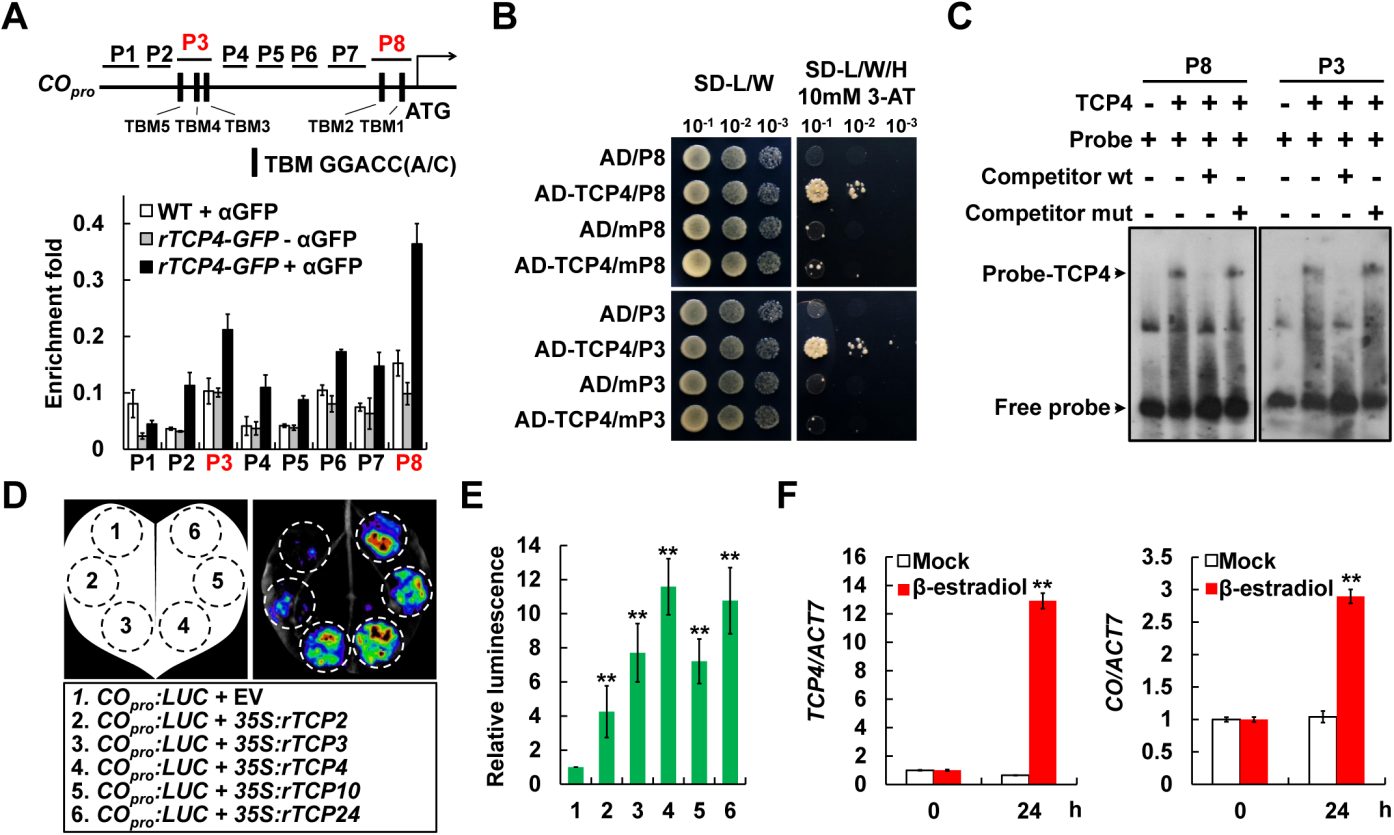
The miR319-regulated TCPs activate *CO* transcription through directly binding to the *CO* promoter. (A) ChIP assays revealing enrichment of TCP4 on the *CO* promoter regions *in vivo*. WT or *BLS*_*pro*_:*rTCP4-GFP* transgenic plants were harvested at ZT 14. Error bars denote ± SD, and three independent biological replicates were performed (n = 3). (B) Yeast one-hybrid (Y1H) assays showing the direct association of TCP4 proteins with *CO* promoter fragments *in vitro*. mP8 and mP3, the fragments which contain mutated TBM *cis*-elements; SD, synthetic dextrose medium; L, Leu; W, Trp; H, His; 3-AT, 3-amino-1,2,4-triazole. 10^−1^, 10^−2^ and 10^−3^ denote the different dilution series. (C) EMSA revealing the binding of TCP4 to the TBM *cis*-elements in *CO* promoter. Competition for TCP4 binding was performed with 125× cold probes containing wild type TBM (Competitor wt) or mutated TBM *cis*-elements (Competitor mut). (D and E) Transcriptional activity assays in *Nicotiana benthamiana* illustrating the activation of *CO* transcription by miR319-targeted TCPs. A representative leaf image was shown in (D), and the quantification of the relative luminescence intensities was done in (E) by using n = 18 independent leaves. Error bars denote SDs. (F) Activation of *CO* transcription in *pERGW-rTCP4* transgenic *Arabidopsis* plants after β-estradiol application. *pERGW-rTCP4* transgenic line was separately treated with DMSO (Mock) or 10 μM β-estradiol at ZT 3, and collected 24 h later for the quantification of the transcript levels of *TCP4* and *CO* (mean ± SD, n = 3). Asterisks in (E) and (F) denote significant differences against the control sample values at *P* < 0.01 (**, Student’s *t* test).

As plant-specific transcription factors, the miR319-regulated TCPs predominantly bind to the common TCP-binding motifs [TBM, GGACC(A/C)] to regulate the expression of target genes [19]. We screened the *CO* promoter sequence (2-kb), and identified five putative TBM sequences. Two of the TBMs are adjacent to the *CO* transcriptional start site (named as TBM 1 and TBM 2) with positions of -263/-257 and -324/-318, while the other three located at -1341/-1335 (named as TBM 3), -1371/-1365 (named as TBM 4) and -1484/-1478 (named as TBM 5), respectively (Fig 3A, upper panel). To investigate the association of TCP4 with the *CO* promoter *in vivo*, we performed chromatin immunoprecipitation (ChIP) assay. Considering that *TCP4* was predominantly expressed in the vascular tissues of leaves (S4 Fig), we used the leaf-expressed and viable *BLS*_*pro*_:*rTCP4-GFP* transgenic plants (labeled as *rTCP4-GFP* in this study; *rTCP4* represents miR319-cleavage-resistant *TCP4*; S5 Fig) [21, 22] for the ChIP assay. Here, we designed eight specific amplicons (represented by P1-P8 in Fig 3A, upper panel) which covered the 2-kb region of *CO* promoter. As a result, the TCP4-GFP enrichments were specifically observed at the P8 and P2/P3/P4 regions of *CO* promoter in the *rTCP4-GFP* ChIP samples (*rTCP4-GFP* + αGFP in Fig 3A), compared with the negative controls (WT + αGFP and *rTCP4-GFP* - αGFP in Fig 3A), with the highest level at the P8 region (Fig 3A). Consistently, P8 and P3 span the *CO* promoter regions where the TCP-binding motifs are located (TBM 1/2 in P8, and TBM 3/4/5 in P3 in Fig 3A), indicating that TCP4 is specifically associated with the TBM *cis*-elements on the *CO* promoter region *in vivo*. This binding specificity was further supported by the yeast one-hybrid (Y1H) and electrophoretic mobility shift (EMSA) assays, in which TCP4 exclusively associated with the P8 and P3 fragments, but not the TBM *cis*-elements mutated mP8 and mP3 [the TBM *cis*-elements GGACC(C/A) were replaced by AAAAAA] (Fig 3B and 3C; S1 and S2 Tables). Together, these data strongly demonstrate that TCP4 directly binds to the TBM *cis*-elements in the *CO* promoter region both *in vitro* and *in vivo*.

Next, to evaluate the direct regulation of TCP4 as well as other miR319-regulated TCPs on *CO* expression, we performed transient transcriptional activity assays in *Nicotiana benthamiana* leaves using the *CO* promoter (2-kb) fused with the *LUC* gene that encodes firefly luciferase as a reporter [23]. Results showed that the LUC signals were significantly elevated by co-expression of the *rTCPs*, including *rTCP2*, *rTCP3*, *rTCP4*, *rTCP10* and *rTCP24* (Fig 3D and 3E; S5 Fig), supporting the hypothesis that the miR319-regulated TCPs are direct transcriptional activators of *CO* transcription. This conclusion was further confirmed in *Arabidopsis* by generating the β-estradiol-inducible *pERGW-rTCP4* transgenic plants (Fig 3F). In the presence of the chemical inducer β-estradiol, the *pERGW-rTCP4* seedlings showed efficient induced expression of *TCP4* (Fig 3F, left panel). Most importantly, the *CO* transcript levels were also significantly up-regulated following the β-estradiol treatment (Fig 3F, right panel), confirming the direct activation of *CO* expression by TCP4.

### The miR319-regulated TCPs physically interact with the flowering activators FBHs but not the flowering repressors CDFs

**Fig 4:**
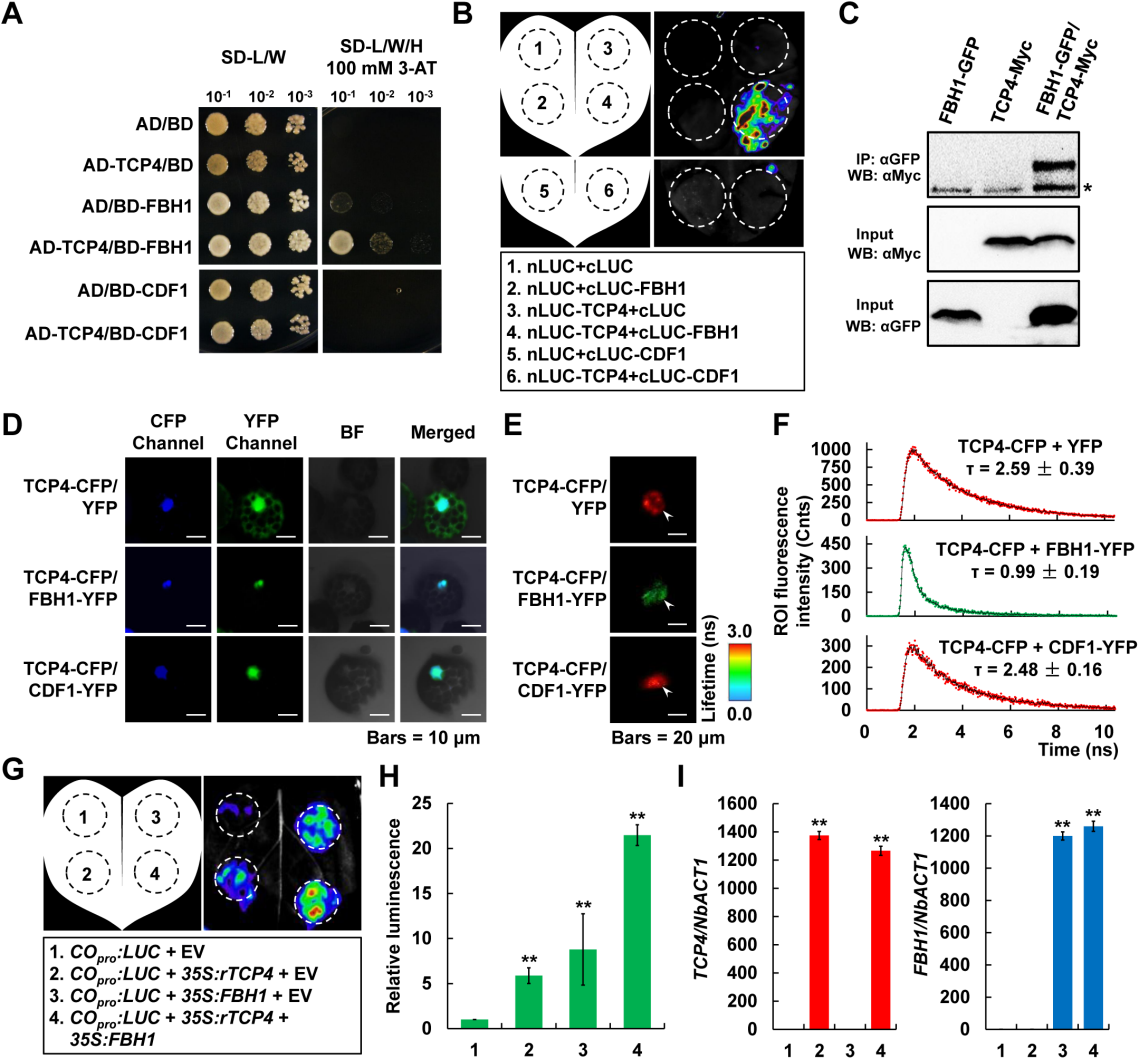
TCP4 physically interacts with FBH1 but not CDF1, and acts additively with FBH1 to activate *CO* transcription. (A) Yeast two-hybrid (Y2H) assay showing the interaction between TCP4 and FBH1. (B) LUC complementation imaging (LCI) assay detecting the interaction among TCP4, FBH1 and CDF1. (C) Co-immunoprecipitation (Co-IP) assay showing the interaction between TCP4 and FBH1 in *Arabidopsis*. The asterisk indicates unspecific signal. (D-F) FLIM-FRET measurement of the interaction between TCP4 and FBH1 in *Arabidopsis* protoplasts. In (D), the CFP and YFP fluorescence signals were observed with the confocal microscope at 24 h after transformation. BF, bright filed; bars = 10 μm. In (E), the white arrowheads indicate the CFP fluorescence lifetime images of the nuclei of representative cells expressing the indicated protein combinations, and the average fluorescence lifetimes are encoded by color as indicated by the scale at right side. Bars = 20 μm. (F) shows the CFP fluorescence intensity decay curves in indicated combinations. The average fluorescence lifetimes represented by τ are separately marked above the curves of TCP4-CFP/YFP (mean ± SD, n = 7 nuclei), TCP4-CFP/FBH1-YFP (mean ± SD, n = 17 nuclei) and TCP4-CFP/CDF1-YFP (mean ± SD, n = 15 nuclei) combinations. ROI, region of interest. (G-I) Transcriptional activity assays in *N*. *benthamiana* illustrating the additive effects of TCP4 and FBH1 in activating *CO* transcription. A representative leaf image is shown in (G), and (H) shows the quantification of the relative luminescence intensities (mean ± SD, n = 18). In (I) the expression levels of *TCP4* and *FBH1* in the infiltrated *N*. *benthamiana* leaf areas were determined by qRT-PCR (mean ± SD, n = 5). Results were normalized to *NbACTIN1* (*NbACT1*). Asterisks in (H) and (I) denote significant differences against the combination 1 at *P* < 0.01 (**, Student’s *t* test).

Because the FBHs and CDFs transcription factors have been shown to separately act as activators and repressors of *CO* transcription [11–13, 24], we asked whether the miR319-regulated TCPs physically interact with these transcription factors. Indeed, yeast two-hybrid (Y2H) assays revealed an obvious interaction between TCP4 and FBH1 (Fig 4A), and this interaction was further confirmed by LUC complementation imaging (LCI) assay in *N*. *benthamiana* (Fig 4B, upper panel). However, no interaction was detected between TCP4 and CDF1 (Fig 4A; Fig 4B, lower panel), suggesting that the interaction between TCP4 and FBH1 may be specifically occurred with biological significance. To further confirm the interaction between TCP4 and FBH1 *in vivo*, we crossed *FBH1-GFP* with *TCP4-Myc* to generate the *FBH1-GFP/TCP4-Myc* double transgenic *Arabidopsis* plant, and conducted co-immunoprecipitation (Co-IP) assay. Confidently, the interaction signal was exclusively observed in *FBH1-GFP/TCP4-Myc* plant, but not in *FBH1-GFP* or *TCP4-Myc* control samples (Fig 4C), further supporting the physical interaction between TCP4 and FBH1. Moreover, we conducted Förster resonance energy transfer (FRET) assays using *Arabidopsis* protoplast cells. Here, we employed a quantitative non-invasive fluorescence lifetime imaging (FLIM) approach to detect FRET efficiency [25]. In this assay, two tested proteins are separately fused with cyan fluorescent protein (CFP) and yellow fluorescent protein (YFP) to generate the donors and acceptors, and the lifetime (described as τ) of the donor fluorescence (CFP) is measured in the presence or absence of the acceptor proteins (represented by τ_DA_ and τ_D_, respectively). If there is a physical interaction between the donor and acceptor, the lifetime τ_DA_ will be considerably shorter than τ_D_. First, we fused TCP4 with CFP to generate the donor, and FBH1 and CDF1 with YFP as the acceptors (Fig 4D–4F). The combination of TCP4-CFP/YFP was also designed to represent the negative control in the absence of acceptor (τ_D_, Fig 4D–4F). Confocal microscope detection suggested that all the indicated proteins were properly accumulated in the protoplast cells, and the fluorescent signals of TCP4-CFP, FBH1-YFP and CDF1-YFP fusion proteins were all exclusively observed and perfectly merged in the nuclei (Fig 4D), indicating similar subcellular localization of TCP4, FBH1 and CDF1. Subsequently, we measured the CFP lifetime in the combination samples. As expected, the average lifetime of CFP in the TCP4-CFP/FBH1-YFP co-expression cells was 0.99 ± 0.19 ns (τ_DA_; mean ± SD, n = 17 nuclei), which was remarkably (*P* < 0.01; Student’s *t* test) shorter than the 2.59 ± 0.39 ns (τ_D_; mean ± SD, n = 7 nuclei) determined in the negative control TCP4-CFP/YFP (Fig 4E and 4F), suggesting a strong physical interaction between TCP4 and FBH1. However, in the TCP4-CFP/CDF1-YFP co-expression samples, the average lifetime of CFP was 2.48 ± 0.16 ns (τ_DA_; mean ± SD, n = 15 nuclei), very similar to that of the negative control, illustrating that TCP4 does not interact with CDF1 (Fig 4E and 4F).

Further assays by Y2H in yeast and LCI in *N*. *benthamiana* revealed that TCP4 also interacts with FBH2, FBH3 and FBH4 (S6A and S6B Fig); meanwhile, FBH1 could also interact with other miR319-regulated TCPs such as TCP2, TCP3, TCP10 and TCP24 (S6C and S6D Fig). These results strongly suggest the functional conservation and redundancy among the miR319-regulated TCPs as well as the FBH homologs. Our simultaneous analyses using the CDF homologs CDF1, CDF2 and CDF3 revealed that these CDFs failed to interact with all the tested TCPs (TCP2, TCP3, TCP4, TCP10 and TCP24) and FBHs (FBH1, FBH2, FBH3 and FBH4) (S7 Fig), indicating that the *CO* transcription repressors CDFs may function independently of the TCPs and FBHs activators.

### TCP4 and FBH1 interact with each other through their transcriptional activation domains and additively activate *CO* transcription

To define the interaction domains between the TCPs and FBHs, we used TCP4 and FBH1 as the representatives in our analyses. First, we generated the different truncated forms of TCP4 and FBH1 (NT, amino terminal; MD, middle domain; CT, carboxyl terminal; S8A Fig). The transcriptional activation activity assay in yeast cells demonstrated that the MD in TCP4 as well as the NT and MD in FBH1 are the functional transcriptional activation domains (S8B Fig). Interestingly, our LCI assays using different truncated forms of TCP4 and FBH1 revealed that the MD of TCP4 and the NT/MD of FBH1 are exactly the domains for their interaction (S8C and S8D Fig). Based on the above analyses, we confidently uncovered the coupling of transcriptional activation domains and their interaction parts between TCP4 and FBH1.

The above conclusion led us to further evaluate the biological significance of the physical interaction between TCP4 and FBH1 on their transcriptional activation activities. To this end, we carried out transient transcriptional activity assays in *N*. *benthamiana*. As the activators of *CO* transcription, both TCP4 and FBH1 significantly led to obvious induction in the luminescence intensity of *CO*_*pro*_:*LUC* by about 6- to 10-fold changes compared with the empty vector control sample (Fig 4G and 4H; combinations 2 and 3). Most importantly, we observed a more significant up-regulation of LUC reporter activity in the TCP4 and FBH1 co-expressed sample (Fig 4G and 4H; combination 4), in which the luminescence intensity was almost 20 times higher than that in the negative control (Fig 4G and 4H; combination 1), confidently suggesting an additive effect of TCP4 and FBH1 on *CO* transcription activation. Meanwhile, our qRT-PCR assays revealed that *TCP4* and *FBH1* were similarly expressed in different infiltrated samples (Fig 4I). Together, these data imply that TCP4 and FBH1 might function additively to regulate the expression of *CO* in *Arabidopsis*.

### Both the miR319-regulated TCPs and FBHs interact with the flowering time regulator PFT1

**Fig 5:**
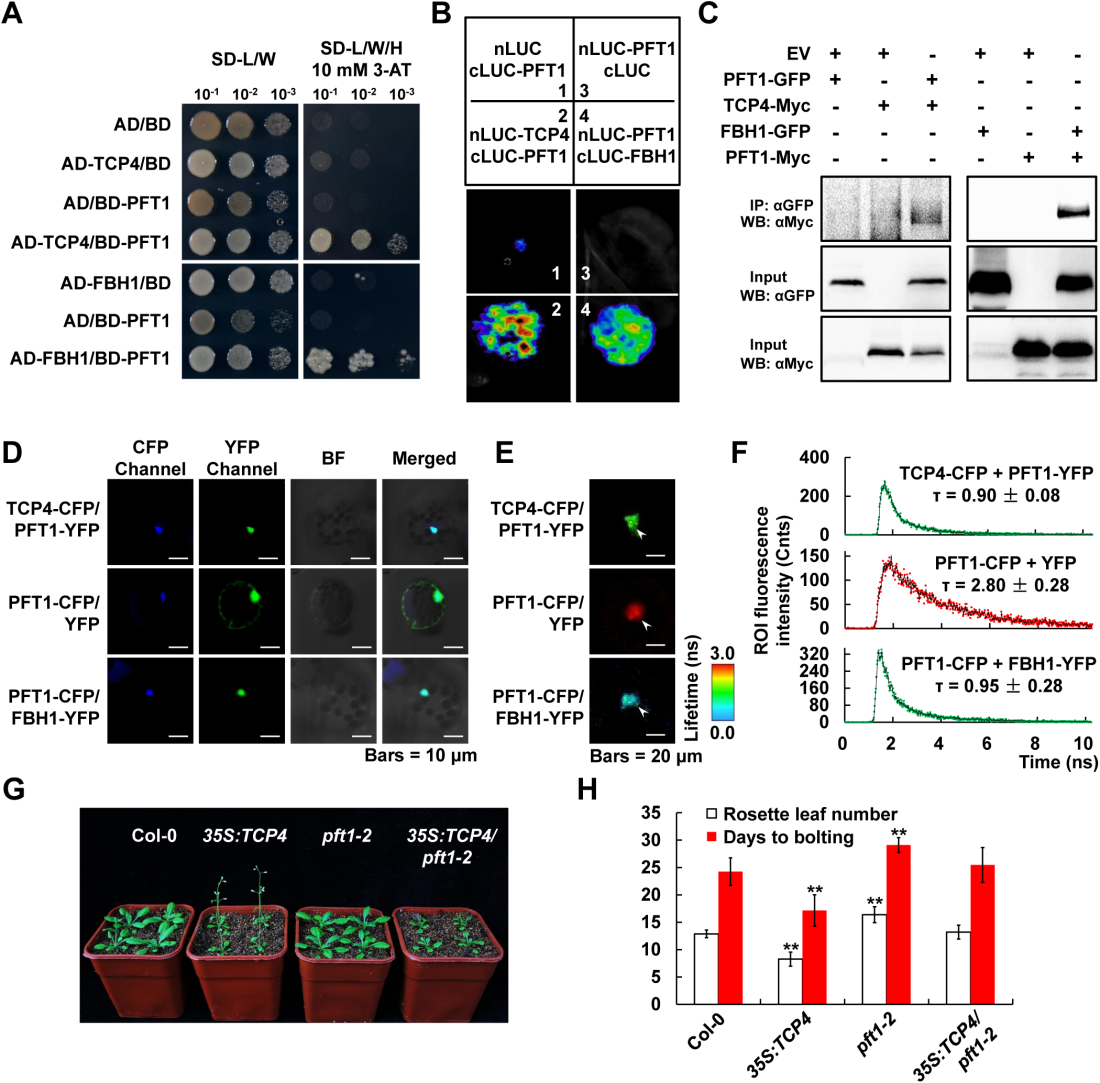
Both TCP4 and FBH1 physically interact with PFT1. (A) Y2H assay showing the interaction of PFT1 with TCP4 and FBH1. (B) LCI assay revealing the interaction of PFT1 with TCP4 and FBH1. (C) Co-IP assay showing the interaction of PFT1 with TCP4 and FBH1. (D-F) FLIM-FRET showing the interaction of PFT1 with TCP4 and FBH1 in *Arabidopsis* protoplasts. The CFP and YFP fluorescence signals were detected in (D). The white arrowheads in (E) indicate the representative CFP fluorescence lifetime images of the nuclei. In (F) the CFP fluorescence intensity decay curves in certain combinations of proteins were shown, and the average fluorescence lifetimes (τ) are marked above the curves of TCP4-CFP/PFT1-YFP (mean ± SD, n = 18 nuclei), PFT1-CFP/YFP (mean ± SD, n = 6 nuclei) and PFT1-CFP/FBH1-YFP (mean ± SD, n = 9 nuclei). (G and H) Genetic interaction of TCP4 and PFT1. The flowering phenotypes of seedlings grown under LDs were shown in (G), and the numbers of rosette leaves (mean ± SD, n ≥ 40) as well as the days to bolting (mean ± SD, n ≥ 15) were calculated in (H).

PFT1, encoding the *Arabidopsis* Mediator complex subunit 25 (MED25) which usually acts as a transcriptional co-activator, was previously described as an essential regulator of flowering time [14, 15]. These observations promoted us to test whether there may be a functional relationship between PFT1 and the *CO* activators TCPs and/or FBHs. Indeed, we found that both TCP4 and FBH1 could interact with PFT1, according to the Y2H assay in yeast as well as the LCI and Co-IP assays in *N*. *benthamiana* (Fig 5A–5C). Next, we conducted FLIM-FRET to further confirm these physical interactions in *Arabidopsis* protoplast cells. Similarly, confocal microscope detection confirmed that the TCP4-CFP, FBH1-YFP and PFT1-YFP/CFP fusion proteins were all properly accumulated, and the fluorescent signals of these fusion proteins were exclusively merged in the nuclei (Fig 5D). Subsequently, an average lifetime of 0.90 ± 0.08 ns (τ_DA_; mean ± SD, n = 18 nuclei) was determined in the TCP4-CFP/PFT1-YFP co-expression samples (Fig 5E and 5F), which was significantly (*P* < 0.01; Student’s *t* test) shorter than that of 2.59 ± 0.39 ns (τ_D_) in the negative control (TCP4-CFP/YFP as shown in Fig 4E and 4F); meanwhile, the average lifetime of CFP in the PFT1-CFP/FBH1-YFP samples was only 0.95 ± 0.28 ns (τ_DA_; mean ± SD, n = 9 nuclei), remarkably (*P* < 0.01; Student’s *t* test) shorter than the 2.80 ± 0.28 ns (τ_D_; mean ± SD, n = 6 nuclei) in the negative control PFT1-CFP/YFP (Fig 5E and 5F). These data strongly demonstrate that PFT1 directly interacts with TCP4 and FBH1 in *Arabidopsis*. Certainly, our extended assays further confirmed that PFT1 also interacts with other miR319-regulated TCPs (including TCP2, TCP3, TCP10 and TCP24) and FBH1 homologs (FBH2, FBH3 and FBH4) (S9 Fig).

### PFT1 Is genetically required for the role of TCP4 in promoting flowering

The above finding that PFT1 physically interacts with TCP4 promoted us to ask whether PFT1 is functionally involved in the TCP4-regulated photoperiodic flowering pathway. To address this question, we crossed the *35S*:*TCP4* transgenic plants with the *pft1-2* mutant line to generate the *35S*:*TCP4/pft1-2* plant, and examined its flowering time phenotype in LDs. Our results showed that *pft1-2* exhibited a late-flowering phenotype compared to the WT Col-0 (Fig 5G and 5H), which is consistent with the previous studies [14, 15, 26]. More importantly, even though *35S*:*TCP4* could trigger early flowering (Fig 1A and 1B; Fig 5G and 5H), its promotional effect on flowering was largely compromised in the *pft1-2* background (Fig 5G and 5H). These results imply that PFT1 is genetically required for the role of TCP4 in promoting flowering.

### PFT1 Acts as co-activator of miR319-regulated TCPs and FBHs to facilitate *CO* transcription

**Fig 6:**
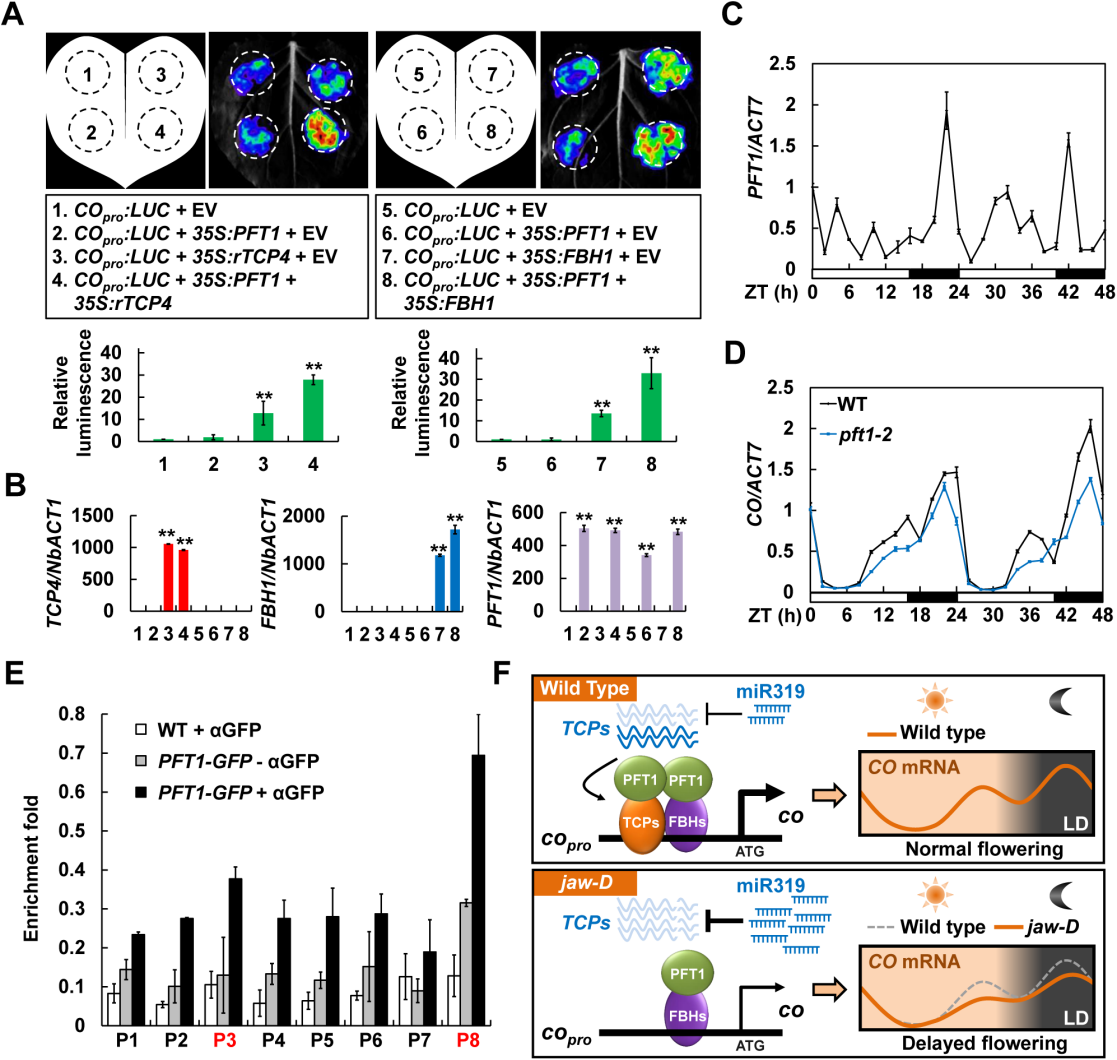
PFT1 facilitates the transcriptional activities of TCP4 and FBH1 in activating *CO* transcription. (A) Transcriptional activity assays in *N*. *benthamiana* showing that PFT1 acts synergistically with TCP4 and FBH1 to activate *CO* transcription. Upper panels show the representative leaf images, and the bottom columns represent the quantification of the relative luminescence intensities (mean ± SD, n = 18). (B) qRT-PCR determination of the expression levels of *TCP4*, *FBH1* and *PFT1* in the infiltrated *N*. *benthamiana* leaf areas shown in (A). Asterisks denote significant differences against the combination 1 or 5 at *P* < 0.01 (**, Student’s *t* test; mean ± SD, n = 5). (C) Temporal expression pattern of *PFT1* in WT under LDs. (D) Temporal expression patterns of *CO* in WT and *pft1-2* mutant line. In (C) and (D), the mean value of WT at ZT 0 was set to 1 (mean ± SD, n = 3). (E) ChIP assays revealing the enrichment of PFT1 on *CO* promoter region *in vivo*. Error bars denote ± SD, n = 3. (F) A proposed working model for photoperiodic flowering control.

We further hypothesized that PFT1 might be required for the full functions of miR319-regulated TCPs in the activation of *CO* transcription. To test this idea, we performed the transient activation activity assays in *N*. *benthamiana* (Fig 6A). Consistent with the above results, the expression of TCP4 alone led to about 10-fold up-regulation of the *CO*_*pro*_:*LUC* reporter activity (Fig 3D and 3E, combination 4; Fig 6A, combination 3); whereas, PFT1 failed to elevate the *CO*_*pro*_:*LUC* reporter activity (Fig 6A, combination 2), indicating that PFT1 alone is not able to activate *CO* transcription. Interestingly, when we co-expressed PFT1 and TCP4, an obvious additive effect was observed as the luminescence intensities increased by almost 30 folds (Fig 6A, combination 4), compared with the empty vector control (Fig 6A, combination 1), significantly higher than that in the TCP4 single-expression samples (Fig 6A, combination 3), suggesting that PFT1 potentially facilitates the transcriptional activation activity of TCP4 on *CO* transcription. Parallel experiments showed that PFT1 could also dramatically enhance the transcriptional activation activity of FBH1 in promoting *CO* transcription (Fig 6A, combinations 5–8). Our qRT-PCR assays revealed that *TCP4*, *FBH1* and *PFT1* were all similarly expressed in different infiltrated samples (Fig 6B). Thus, we concluded that PFT1 may function as co-activator of both TCPs and FBHs in the activation of *CO* transcription.

### PFT1 is enriched in the *CO* promoter to positively regulate *CO* transcription

To well understand the action mechanism by which PFT1 regulates flowering time, we first analyzed the time-course expression pattern of *PFT1* under LDs. Interestingly, similar to *CO* [2, 27], *PFT1* displayed a diurnal rhythmic expression pattern, with a small elevation of *PFT1* mRNA levels in the afternoon (ZT 8–12) and an obvious peak at the midnight (ZT 20–24) (Fig 6C). In consistence with the previous report [14], our data confirmed that the *CO* expression in the *pft1-2* mutant line was significantly compromised compared with that in the WT Col-0 control (Fig 6D), indicating an essential role of PFT1 in facilitating *CO* transcription.

Based on our findings that PFT1 physically interacts with the *CO* activators TCPs and FBHs (Fig 4), we were interested to test whether PFT1 proteins are enriched in the *CO* promoter regions *in vivo*. To this end, we conducted the ChIP assays using the *35S*:*PFT1-GFP* transgenic *Arabidopsis* plants [15]. As expected, the PFT1-GFP enrichments were remarkably detected at the P8 and P3 regions of *CO* promoter with a maximum enrichment at P8 (*PFT1-GFP* + αGFP in Fig 6E), compared with the negative controls (WT + αGFP and *PFT1-GFP* - αGFP in Fig 6E). Significantly, the enrichment tendency of PFT1 at the different *CO* promoter regions well correlated with that of TCP4 (Fig 3A), and was also very similar to that of FBH1 as shown in a previous study [13]. Taken together, we propose that PFT1 is probably enriched in the *CO* promoter regions to act synergistically with TCPs and FBHs to facilitate *CO* transcription.

## Discussion

In this study, we showed that the miR319-regulated TCPs interact with the flowering time regulators FBHs and PFT1 to activate *CO* transcription and promote *Arabidopsis* photoperiodic flowering.

Previous observations suggested that the miR319-regulated TCPs transcription factors may be involved in regulation of *Arabidopsis* flowering time [17–19]. However, the functional mode and action mechanism of these TCPs transcription factors in regulation of *Arabidopsis* flowering time remain unclear. In this study, we show that down-regulation of the miR319-regulated *TCPs* in the *jaw-D* mutant plants causes late flowering phenotype in LDs, but not in SDs (Fig 1A and 1B; S1 Fig), demonstrating that the miR319-regulated TCPs modulate flowering time through regulating the photoperiodic flowering pathway in *Arabidopsis*. In support of this view, the expression of *CO*, a central component of the photoperiodic flowering pathway in *Arabidopsis*, were significantly reduced in the *jaw-D* mutant plants in amplitude under LDs (Fig 2A), while up-regulated by both constitutive and inducible overexpression of *TCP4* (Figs 2A and 3F). Further, we showed that TCP4 can bind to the TBM *cis*-elements of the *CO* promoter and all the miR319-regulated TCPs directly activate *CO* transcription (Fig 3A–3E). Based on these findings, we conclude that the miR319-regulated TCPs may act as positive regulators of photoperiodic flowering through direct activation of *CO* transcription in *Arabidopsis*. Nevertheless, the *in planta* interplay between the miR319-regulated TCP transcription factors and *CO* promoter still needs to be intensively analyzed in the future, considering that the non-native promoter used for driving *TCP4* expression in this study might cause a non-physiological effect for TCP4. Therefore, it should be intriguing to uncover the dynamic enrichment pattern of each member of these TCP proteins on the *CO* promoter, which will be useful for better understanding the contribution of these TCPs to the daily *CO* oscillation.

FBHs act as *CO* transcription activators in regulating flowering time [13]. Our findings that TCPs physically interact with FBHs provide a novel mechanism for the regulation of *CO* transcription in the photoperiodic flowering pathway (Fig 4; S6 and S8 Figs). The previous study showed that the E-box *cis*-elements contained in the -509/-196 region of *CO* promoter are essential for FBH1 binding as well as FBH1-dependent gene activation [13]. Coincidently, our ChIP assays revealed a preferred binding fragment of *CO* promoter by TCP4 containing the TBM *cis*-elements in the -348/-155 region (P8 in Fig 3A), which is adjacent to and partially overlapped with the FBH1 binding region. The spatial proximity of the DNA-binding sites to some extent causes the possibility of direct interaction between TCP4 and FBH1. However, our further assays revealed that TCP4 and FBH1 interact with each other through their transcriptional activation domains (S8 Fig), not through their DNA-binding domains (i.e. the bHLH domains that located in the N- or C-terminals of TCP4 and FBH1, respectively, as shown in S8A Fig), suggesting the physical interaction between TCP4 and FBH1 might facilitate their transcriptional activation activities on *CO* transcription. Indeed, an additive effect of TCP4 and FBH1 in activating *CO* transcription was obviously observed in our analyses (Fig 4G and 4H), implying a potential interplay among the TCPs and FBHs transcription factors. However, it should be noticed that TCP4 and FBH1 themselves could, at least in part, activate the transcription of *CO* (Fig 4G and 4H). Thus, the additive effect of TCP4 and FBH1 might be attributed to more abundant activators enriched on the *CO* promoter and/or their cooperation upon the co-expression of these two transcription factors. Here, we assume that the miR319-regulated TCPs and FBHs might function cooperatively and/or independently to activate the *CO* expression in certain situations. However, it is eagerly needed to explore the genetic interaction between the TCPs and FBHs regarding the regulation of *CO* expression *in vivo* in the future.

In this study, we confirmed that both TCPs and FBHs physically interact with the transcriptional co-activator PFT1 (Fig 5 and S9 Fig). Although PFT1, encoding the Mediator subunit 25 in *Arabidopsis*, was initially identified as a positive regulator of flowering time more than ten years ago [14], the molecular mechanisms of its action in regulation of flowering time remain obscure to date. Mediator is a multiprotein complex that promotes transcription by recruiting the RNA polymerase II (RNAPII) to the promoter regions upon the physical interaction with specific DNA-bound transcription factors [28, 29]. Our observations reinforce that co-expression of PFT1/MED25 with TCP4 or FBH1 additively elevated the *CO* transcription levels (Fig 6A), while the loss-of-function of *PFT1* leads to an obvious reduction of *CO* mRNA levels (Fig 6D). It is noteworthy, in our assays, that PFT1 failed to promote *CO* transcription in the absence of TCP4 or FBH1 (Fig 6A), implying the essential roles of TCPs and FBHs for the function of PFT1 in activating *CO* transcription. This hypothesis was further supported by our ChIP assay results that the PFT1 proteins were enriched with the peaks in the *CO* promoter regions near the TCP4- and FBH1-binding sites (Figs 3A and 6E). Collectively, our results suggest that PFT1 potentially acts as positive regulator of *CO* transcription.

Based on our findings, we proposed a working model on the control of photoperiodic flowering time (Fig 6F). Briefly, the miR319-regulated TCPs and FBHs directly bind to the adjacent regions of *CO* promoter in the wild-type *Arabidopsis* plants; they physically interact with each other through their transcriptional activation domains to activate *CO* transcription through direct interaction with PFT1, and consequently promote flowering under LDs (Fig 6F, upper panel). By contrast, in the *jaw-D* mutant plants, the association of TCPs with *CO* promoter is drastically blocked due to the overdose of miR319 and consequent decrease of TCP proteins, leading to down-regulation of *CO* transcription during the peak expression time, which as a result causes delayed flowering (Fig 6F, lower panel).

## Materials and methods

### Plant materials and growth conditions

The transgenic and mutant lines used in this study were previously described: *jaw-1D* [17]; *co-9* [20]; *BLS*_*pro*_:*rTCP4-GFP* [21]; *CO*_*pro*_:*GUS* [30]; *35S*:*CO* [7]; *pft1-2* [26]; *35S*:*PFT1-GFP* [15].

*Arabidopsis thaliana* were grown under LD (16-h-light/8-h-dark) or SD (8-h-light/16-h-dark) conditions at 22°C. Time-course analyses were performed on 12-d-old seedlings grown on half-strength Murashige and Skoog medium. *Nicotiana benthamiana* was grown in a greenhouse at 22°C with a 16-h-light/8-h-dark cycle.

### Analyses of flowering time phenotype

Analyses of flowering time were performed as previously described [15]. Flowering time was recorded from at least 15 plants per genotype that were grown in soil under either LDs or SDs, and was scored as the number of days from germination to the first appearance of buds at the apex (days to bolting). The rosette leaf number was counted after the main stem has bolted 1 cm.

### DNA constructs and generation of transgenic/hybrid plants

For Gateway cloning, all the gene sequences were cloned into the pQBV3 or pENTRY vectors (Gateway) and subsequently introduced into certain destination vectors following the Gateway technology (Invitrogen). For ligase dependent cloning, the endonuclease digested vectors and PCR fragments were separately purified by PCR cleanup kit (Axygen, AP-PCR-250), and ligated at 16°C with T4 DNA ligase (New England Biolabs, M2020). For ligase-independent ligation, the ligation free cloning mastermix (abm) was used following the application handbook.

For generation of miR319-cleavage-resistant forms of *TCPs* (*rTCPs*) as well as the TBM *cis*-elements mutated *CO* promoter fragments, the one-step site-directed mutagenesis strategy was performed. The wild type *TCP* (*TCP2*, *TCP3*, *TCP4*, *TCP10* and *TCP24*) sequences or the *CO* promoter fragments were first connected into the pQBV3 entry vector, and then the mutations on miR319-target sites or TBM *cis*-elements were introduced by the specifically designed primers, as described previously [31]. The PCR amplifications were carried out by pre-heating at 94°C for 3 min, 16 cycles of 94°C for 1 min, 55°C for 1 min and 68°C for 7 min, followed by incubation at 68°C for 1 h. The PCR products were purified by PCR cleanup kit (Axygen, AP-PCR-250), and digested by 1 μl of DpnI (New England Biolabs, R0176L). The obtained products were transformed into *Escherichia coli* competent cells for sequencing. The primer sequences used for site-directed mutagenesis are listed in S5 Table.

For the generation of *35S*:*TCP4* lines, the expression vector *35S*:*TCP4* [17] was transformed into the *Agrobacterium* strain GV3101 (pMP90). For the construction of *TCP4*_*pro*_:*GUS* transgenic lines, the 5’ upstream region of the *TCP4* sequence (-2000/-1) was amplified from Col-0 genomic DNA, and cloned into the binary vector pMDC162 vector to generate *TCP4*_*pro*_:*GUS* expression construct. For the generation of β-estradiol-inducible *pERGW-rTCP4* transgenic plants, the construct *pQBV3-rTCP4* containing the miR319-cleavage-resistant *TCP4* was introduced into the destination vector pERGW to fuse with a β-estradiol-inducible promoter following the Gateway cloning strategy. All of the binary vectors were introduced into the wild type Col-0 plants by *Agrobacterium*-mediated transformation to generate transgenic plants [20]. More details of the DNA constructs are listed in S6 Table. *jaw-D/35S*:*CO*, *co-9/35S*:*TCP4*, *FBH1-GFP/TCP4-Myc* and *35S*:*TCP4/pft1-2* were generated by genetic crossing.

### RNA extraction and gene expression analysis

The 12-d-old *Arabidopsis* seedlings grown on half-strength Murashige and Skoog medium were collected at the indicated time after the onset of light. Total RNA was extracted using Trizol (Invitrogen) reagent. About 2 μg of total RNA and Moloney murine leukemia virus reverse transcriptase (M-MLV; Promega) were further used for reverse transcription. The cDNA was diluted to 100 μL with water in a 1:5 ratio, and 2 μL of the diluted cDNA was used for quantitative reverse transcription-polymerase chain reaction (qRT-PCR) with the SYBR Premix Ex Taq (Perfect Real Time; TaKaRa). The reverse transcription of mature miRNAs was performed as described previously [32], by using specifically designed stem-loop primers. qRT-PCR was performed using the following program: 120 sec at 95°C, 45 cycles of 10 sec at 95°C, and 1 min at 60°C. *ACTIN7* and *U6* expression levels were used as the internal controls for coding genes and miRNAs, respectively. All the experiments were performed independently three times. All the primers used for real time quantification are listed in S3 Table.

### Yeast experiments

For yeast one-hybrid assay, the pHIS2 derivatives were co-transformed with GAL4-AD constructs harboring the P8 and P3 sequences, or mP8 and mP3 fragments which contain mutated TBM *cis*-elements [GGACC(C/A) were replaced by AAAAAA] (see S1 Table) into the yeast (*Saccharomyces cerevisiae*) strain AH109. The transformed cells were first grown on synthetic dextrose medium lacking Leu and Trp (SD-L/W) and then were transferred to the synthetic dextrose medium lacking Leu, Trp and His (SD-L/W/H) medium supplemented with 3-amino-1,2,4-triazole (3-AT) for selection. For yeast two-hybrid assay, the GAL4-AD and GAL4-BD derivatives were co-transformed into the yeast strain AH109, and grown on SD-L/W. Further, the yeast cells were screened on the SD-L/W/H or SD-L/W/H/A media with 3-AT. For transcriptional activation activity analysis, the GAL4-BD derivatives were separately transformed into yeast strain AH109, and the transformed yeast cells were grown and selected on SD-L and SD-L/H/A media, respectively. Each experiment was independently repeated for three times with similar results.

### LCI assays

The LCI assays for the protein interaction detection was performed in *N*. *benthamiana* leaves as described previously [33]. Briefly, the full-length or truncated forms of the genes were separately fused with the N- and C-terminal parts of the luciferase reporter gene *LUC*. *Agrobacteria* cells harboring the nLUC and cLUC derivative constructs were co-infiltrated into *N*. *benthamiana* leaves, and the LUC activities were analyzed 48 h after infiltration using NightSHADE LB 985 (Berthold). In each analysis, five independent *N*. *benthamiana* leaves were infiltrated and analyzed, and totally three biological replications were performed with similar results.

### Transcriptional activity assays in *N*. *benthamiana*

The transcriptional activity assays were performed in *N*. *benthamiana* leaves as previously described [23]. The 2-kb *CO* promoter sequence was amplified from Col-0 genome DNA, and fused with the luciferase reporter gene *LUC* through Gateway reactions (Invitrogen) into the plant binary vector pGWB35 [34] to generate the reporter construct *CO*_*pro*_:*LUC*. For the construction of the effectors, the full-length coding sequences of indicated genes were amplified and cloned into the plant binary vector pGWB17 [34]. The reporter and effector constructs were separately introduced into *Agrobacterium* strain GV3101 (pMP90), to carry out the co-infiltration in *N*. *benthamiana* leaves. LUC activities were observed and quantified 48 h after infiltration using NightSHADE LB 985 (Berthold). In each experiment, 10 independent *N*. *benthamiana* leaves were infiltrated and analyzed, and totally three biological replications were performed with quantification.

### Protein extraction and co-immunoprecipitation analysis

The co-immunoprecipitation (Co-IP) analyses were performed using transgenic *Arabidopsis* seedlings or *Agrobacterium*-infiltrated *N*. *benthamiana* leaves. The indicated genes were combined into pGWB5 and pGWB17 vectors to produce the GFP- or Myc-fused constructs, and transformed into *Agrobacterium* strain GV3101. For *Agrobacterium*-mediated transient expression, the *Agrobacteria* harboring indicated derivatives were co-infiltrated into the *N*. *benthamiana* leaves, and the samples were collected 48 h post infiltration. The total proteins were extracted using the lysis buffer (50 mM Tris-HCl at pH 7.5, 150 mM NaCl, 5 mM EDTA at pH 8.0, 0.1% Triton X-100, 0.2% NP-40) with freshly added PMSF (phenylmethylsulphonyl fluoride, 10 mM) and protease inhibitor cocktail (Roche, 11873580001). Anti-GFP- and Anti-Myc-conjugated agarose beads (MBL, M047-8 and D153-8) were used for the immunoprecipitation. In western blotting, anti-GFP (1:2000; Roche, 11814460001), anti-Myc (1:5000; Roche, 11667149001) and anti-mouse IgG (1:75000, Sigma, A9044-2ML) antibodies were used for the detection of GFP- and Myc-tagged proteins. Totally three independent biological replicates were performed.

### FLIM-FRET assays

For FLIM-FRET assays, the indicated proteins were separately fused with cyan fluorescent protein (CFP) and yellow fluorescent protein (YFP) to generate the donors and receptors. The FLIM-FRET experiments were performed as described previously [25] with some modifications. Briefly, the donors and receptors were co-expressed in *Arabidopsis* protoplast cells. After 24 h incubation, the CFP and YFP fluorescence signals were imaged with the confocal microscope (Carl Zeiss, LSM880). Förster resonance energy transfer was measured by fluorescence lifetime imaging using the PicoHarp 300 time-correlated single-photon counting (TCSPC) module (PicoQuant, Germany). A pulsed laser (405 nm) tuned at 84MHz was used to excite the CFP. The emission from 450 to 490 nm was collected by the detector in 512×512 pixel format. The acquired FLIM decay curve from regions of interest (ROI) was fitted by two-exponential theoretical models using SymPhoTime 64 software, and the mean CFP lifetimes were calculated as the mean values of the fit function and analyzed using SymPhoTime 64 software. In each analysis, 6 independent nuclei were quantitatively analyzed, and totally three independent replications were performed with similar results.

### ChIP assays

*Arabidopsis* seedlings were grown on half-strength Murashige and Skoog medium in 16-h-light/8-h-dark LDs for 14 days, and the samples (2 to 3 grams) were collected at ZT 14. The ChIP assays were carried out following the procedure described previously [23]. The ChIP assays were separately performed with (+ αGFP) or without (- αGFP) the anti-GFP antibody. Finally, the GFP-specific enrichments of the fragments from *CO* promoter were analyzed by qPCR, and the enrichment fold of a certain fragment was calculated by normalizing to the amount of *ACTIN7* promoter enriched in the same sample. Anti-GFP-CHIP grade antibody (Abcam, ab290) and protein G plus agarose (Santa cruz, sc-2002) were used for the immunoprecipitation. The enrichment of DNA fragments was determined by qPCR with specific primers, as shown in S4 Table. Three biological replicates were performed.

### EMSA

The maltose binding protein (MBP) tagged TCP4 protein was expressed in *E*. *coli* strain *Tran*setta-DE3 (Transgen biotech, CD801), and purified using the amylose resin (New England Biolabs, E8021V) following the manual. The *CO* promoter probes containing the TBM *cis*-element were synthesized and labeled with digoxigenin-11-ddUTP at the 3’ end by using DIG gel shift kit (Roche, 03353591910). Unlabeled dimerized oligo-nucleotides of *CO* promoter fragments containing the wild type or mutated TBM *cis*-elements were generated as the competitors. EMSAs were performed as previously described [33]. Competition for TCP4 binding was performed with 125× cold probes containing TBM *cis*-elements [GGACC(C/A)] or mutated TBM *cis*-elements (AAAAAA). Sequences of probes and competitors are shown in S2 Table. Three biological replicates were performed.

### Accession numbers

Sequence data from this study can be found in the *Arabidopsis* Genome Initiative database under the following accession numbers: *CO* (At5g15840), *TCP2* (At4g18390), *TCP3* (At1g53230), *TCP4* (At3g15030), *TCP10* (At2g31070), *TCP24* (At1g30210), *FT* (AT1G65480), *FBH1* (At1g35460), *FBH2* (At4g09180), *FBH3* (At1g51140), *FBH4* (At2g42280), *CDF1* (At5g62430), *CDF2* (At5g39660), *CDF3* (At3g47500), *PFT1* (At1g25540), *ACTIN7* (At5g09810) and *U6* (At3g14735).

## Supporting information

S1 Fig Flowering times of the indicated genotypes under SDs. The numbers of rosette leaves (mean ± SD, n ≥ 15) as well as the days to bolting (mean ± SD, n ≥ 15) of Col-0, *jaw-D* and *35S*:*TCP4* were separately counted. SD, short-day (8 h light/16 h dark) condition.
(TIF)

S2 Fig Expression of *TCP4* and *CO* in Col-0 and *35S*:*TCP4* transgenic plants. The 12-d-old seedlings of wild type (WT) Col-0 and *35S*:*TCP4* transgenic plants were harvested at *Zeitgeber* time (ZT) 3, and the expression levels of *TCP4* (A) and *CO* (B) in WT and *35S*:*TCP4* were separately quantified by qRT-PCR. All the values were normalized to the internal control gene *ACT7* (mean ± SD, n = 3), and the mean values in WT Col-0 were set to 1. Asterisks denote significant differences compared with the WT negative control at *P* < 0.01 (**, Student’s *t* test).
(TIF)

S3 Fig *FT* expression levels in *jaw-D* and *35S*:*TCP4* plants. The 12-d-old *Arabidopsis* seedlings of Col-0, *jaw-D* and *35S*:*TCP4* were separately collected at ZT 0, 6, 12 and 18. All the values were normalized to the internal control genes *ACT7* (mean ± SD, n = 3). The mean value in WT Col-0 at ZT 0 was set to 1. Asterisks above the bars denote significant differences at *P* < 0.01 (**, Student’s *t* test).
(TIF)

S4 Fig Spatial expression pattern of *TCP4* in WT plant. The whole-mount staining of a 7-d-old seedling (A), a 12-d-old seedling (B), a cotyledon (C) and the first set of true leaf (D) from the plants carrying the *TCP4*_*pro*_:*GUS* (β-glucuronidase) reporter gene were shown with scale bars (1 mm).
(TIF)

S5 Fig The partial mRNA sequences of the wild type and miR319-cleavage-resistant derivatives of miR319-regulated *TCP* genes. The sequences of three miR319 members from *Arabidopsis* are shown above, and the single nucleotide variant in miR319c is marked by the purple color. The nucleotide mutations were introduced into the miR319 target site in *TCPs* to produce miR319-cleavage-resistant *rTCPs* without changing the coded protein sequences. The synonymous changes are marked by red color, and the partial coded protein sequences are shown below.
(TIF)

S6 Fig Physical interaction between miR319-regulated TCPs and FBH homologs. (A and B) Y2H and LCI detections of the interaction between TCP4 and FBHs, including FBH2, FBH3 and FBH4. (C and D) Y2H and LCI assays showing the interaction between FBH1 and TCPs (TCP2, TCP3, TCP10 and TCP24). SD-L/W, synthetic dextrose medium lacking Leu and Trp; SD-L/W/H/A, synthetic dextrose medium lacking Leu, Trp, His and Ade; 3-AT, 3-amino-1,2,4-triazole; 10^−1^, 10^−2^, 10^−3^ and 10^−4^ denote the different dilution series.
(TIF)

S7 Fig Neither the miR319-regulated TCPs nor FBHs interact with CDFs. (A) Y2H assays to determine the interactions among TCPs-CDFs and FBHs-CDFs (n = 3). SD-L/W, synthetic dextrose medium lacking Leu and Trp; SD-L/W/H/A, synthetic dextrose medium lacking Leu, Trp, His and Ade; 3-AT, 3-amino-1,2,4-triazole; 10^−1^, 10^−2^, 10^−3^ and 10^−4^ denote the different dilution series. (B) LCI assays to detect the interaction between TCP4 and CDFs transcription factors (n = 5). (C) LCI assays to detect the interaction between FBH1 and CDFs transcription factors (n = 5).
(TIF)

S8 Fig TCP4 and FBH1 interact with each other through their transcriptional activation domains. (A) Schemes display full length structures of TCP4 and FBH1 proteins as well as truncated versions. The locations of bHLH domains in TCP4 and FBH1 are marked by black and blue boxes, respectively. NT, amino terminal; MD, middle domain; CT, carboxyl terminal. Scale bar = 50 amino acids (aa). (B) Determination of the transcriptional activation domains of TCP4 and FBH1 in yeast. SD-L, synthetic dextrose medium lacking Leu; SD-L/H/A, synthetic dextrose medium lacking Leu, His and Ade; 10^−1^, 10^−2^, 10^−3^ and 10^−4^ denote the different dilution series. (C) LCI assay showing the interaction between truncated TCP4 versions and the full length FBH1. (D) LCI assay showing the interaction between full length TCP4 and the truncated FBH1 versions. The LUC signals in (C) and (D) were collected at 48 hpi (n = 5).
(TIF)

S9 Fig Both the miR319-regulated TCPs and FBHs interact with PFT1. (A) Y2H assay showing the physical interaction between PFT1 and TCPs/FBHs (n = 3). (B) LCI assay showing the interaction between PFT1 and the miR319-regulated TCPs (n = 5). (C) LCI assay showing the interaction between PFT1 and FBHs (n = 5).
(TIF)

S1 Table *CO* promoter fragments used in yeast one-hybrid assay. (DOCX)

S2 Table Probes used in EMSA experiment. (DOCX)

S3 Table Primers used for qRT-PCR. (DOCX)

S4 Table Primers used for ChIP-qPCR assays. (DOCX)

S5 Table Primers used for site-directed mutagenesis. (DOCX)

S6 Table Constructs used in this study. (DOCX)
