# Supplementary figures and images for: MicroRNA319-regulated TCPs interact with FBHs and PFT1 to activate CO transcription and control flowering time in Arabidopsis
Source: PLoS Genet. 2017 May 30;13(5):e1006833. doi: 10.1371/journal.pgen.1006833 (PMC5469495; doi:10.1371/journal.pgen.1006833)

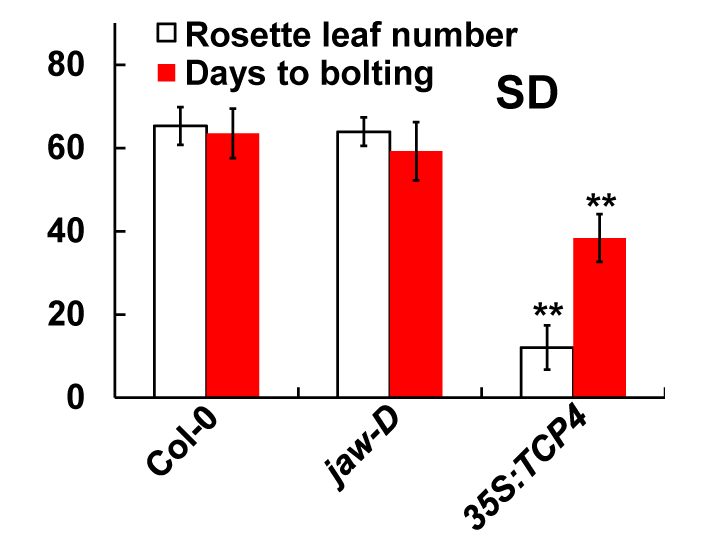

Supplement: S1 Fig — The numbers of rosette leaves (mean ± SD, n ≥ 15) as well as the days to bolting (mean ± SD, n ≥ 15) of Col-0, jaw-D and 35S:TCP4 were separately counted. SD, short-day (8 h light/16 h dark) condition. (TIF) [file pgen.1006833.s001.tif]

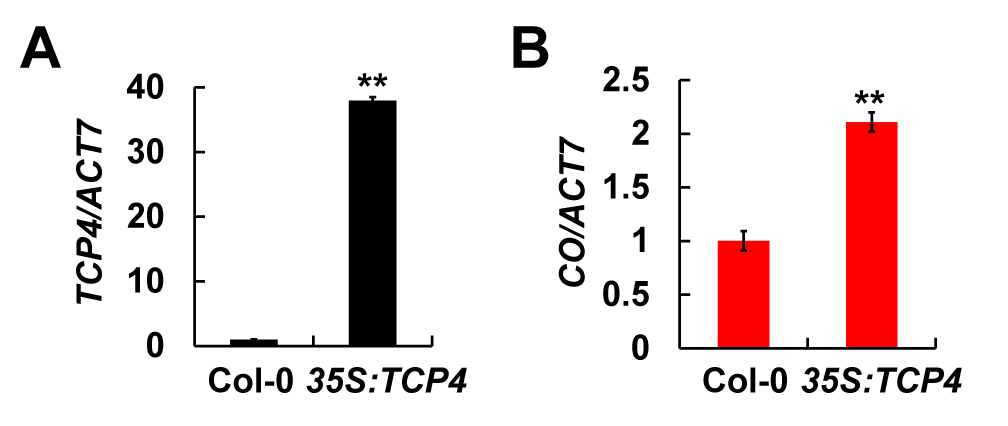

Supplement: S2 Fig — The 12-d-old seedlings of wild type (WT) Col-0 and 35S:TCP4 transgenic plants were harvested at Zeitgeber time (ZT) 3, and the expression levels of TCP4 (A) and CO (B) in WT and 35S:TCP4 were separately quantified by qRT-PCR. All the values were normalized to the internal control gene ACT7 (mean ± SD, n = 3), and the mean values in WT Col-0 were set to 1. Asterisks denote significant differences compared with the WT negative control at P < 0.01 (**, Student’s t test). (TIF) [file pgen.1006833.s002.tif]

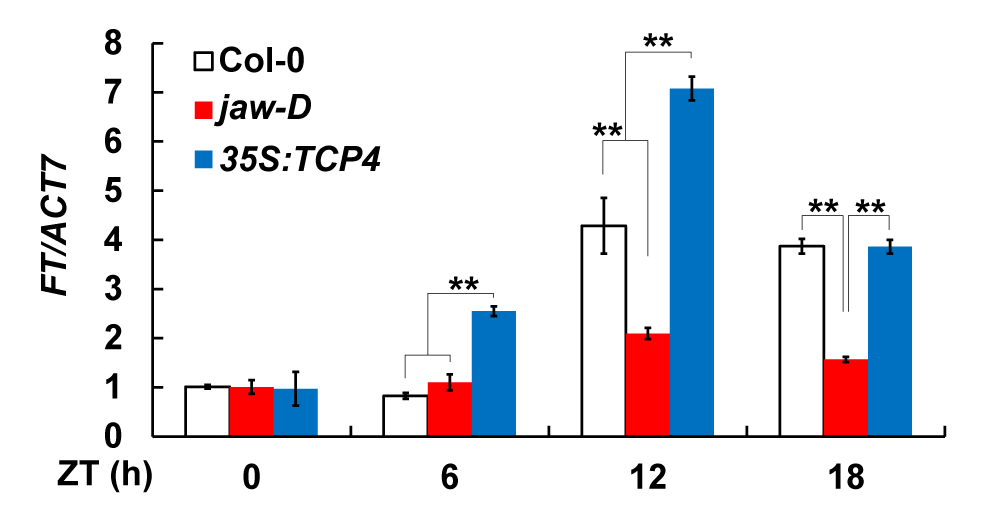

Supplement: S3 Fig — The 12-d-old Arabidopsis seedlings of Col-0, jaw-D and 35S:TCP4 were separately collected at ZT 0, 6, 12 and 18. All the values were normalized to the internal control genes ACT7 (mean ± SD, n = 3). The mean value in WT Col-0 at ZT 0 was set to 1. Asterisks above the bars denote significant differences at P < 0.01 (**, Student’s t test). (TIF) [file pgen.1006833.s003.tif]

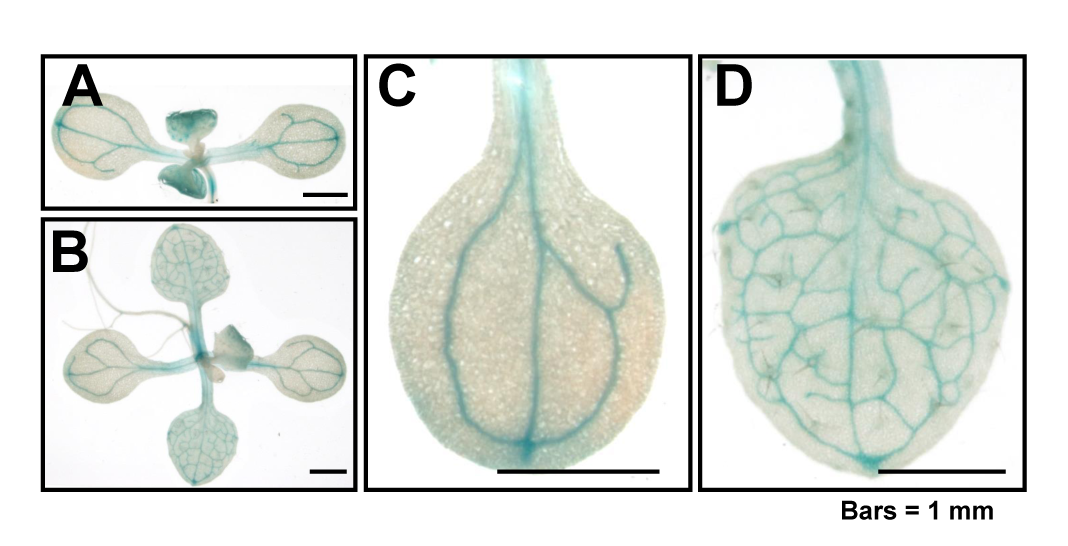

Supplement: S4 Fig — The whole-mount staining of a 7-d-old seedling (A), a 12-d-old seedling (B), a cotyledon (C) and the first set of true leaf (D) from the plants carrying the TCP4pro:GUS (β-glucuronidase) reporter gene were shown with scale bars (1 mm). (TIF) [file pgen.1006833.s004.tif]

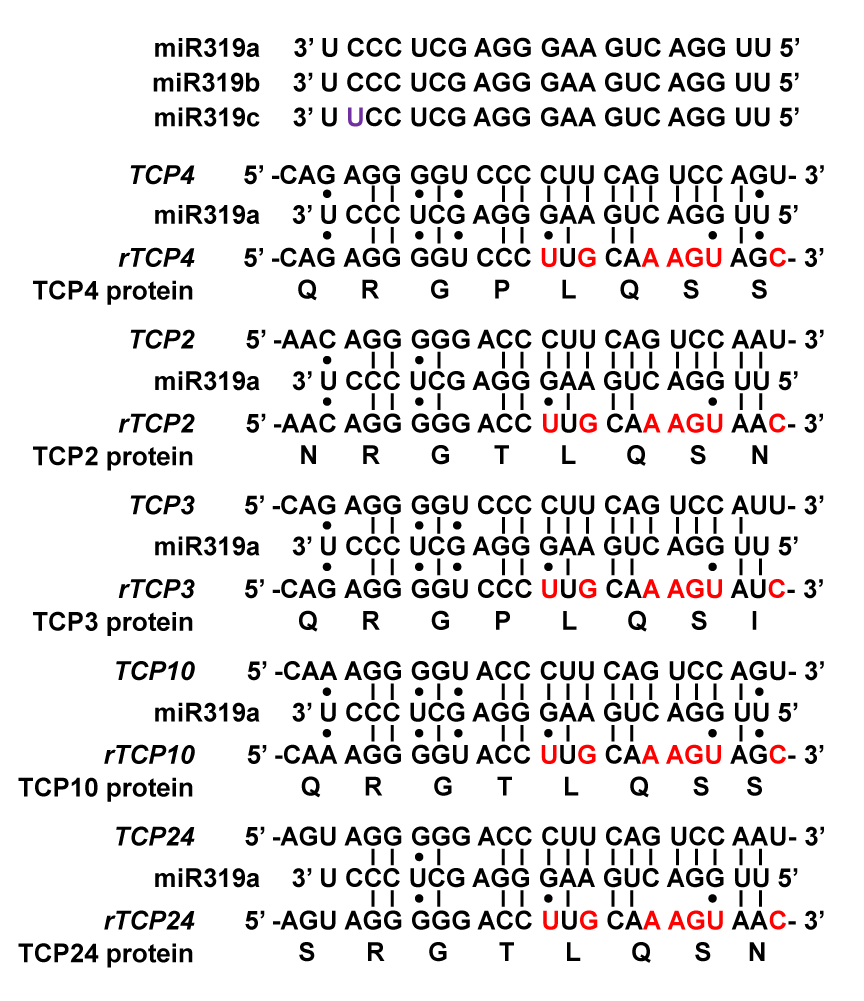

Supplement: S5 Fig — The sequences of three miR319 members from Arabidopsis are shown above, and the single nucleotide variant in miR319c is marked by the purple color. The nucleotide mutations were introduced into the miR319 target site in TCPs to produce miR319-cleavage-resistant rTCPs without changing the coded protein sequences. The synonymous changes are marked by red color, and the partial coded protein sequences are shown below. (TIF) [file pgen.1006833.s005.tif]

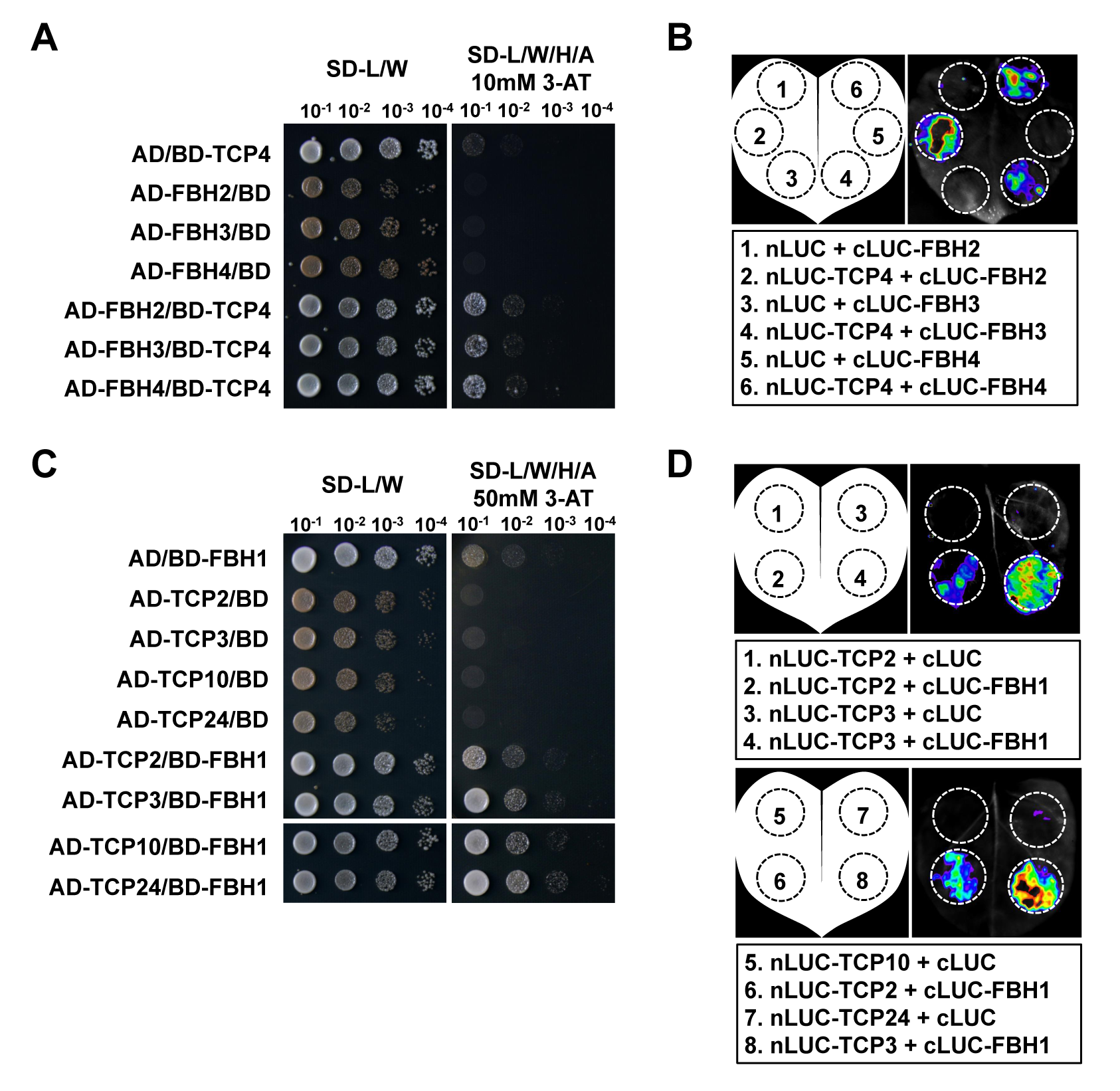

Supplement: S6 Fig — (A and B) Y2H and LCI detections of the interaction between TCP4 and FBHs, including FBH2, FBH3 and FBH4. (C and D) Y2H and LCI assays showing the interaction between FBH1 and TCPs (TCP2, TCP3, TCP10 and TCP24). SD-L/W, synthetic dextrose medium lacking Leu and Trp; SD-L/W/H/A, synthetic dextrose medium lacking Leu, Trp, His and Ade; 3-AT, 3-amino-1,2,4-triazole; 10−1, 10−2, 10−3 and 10−4 denote the different dilution series. (TIF) [file pgen.1006833.s006.tif]

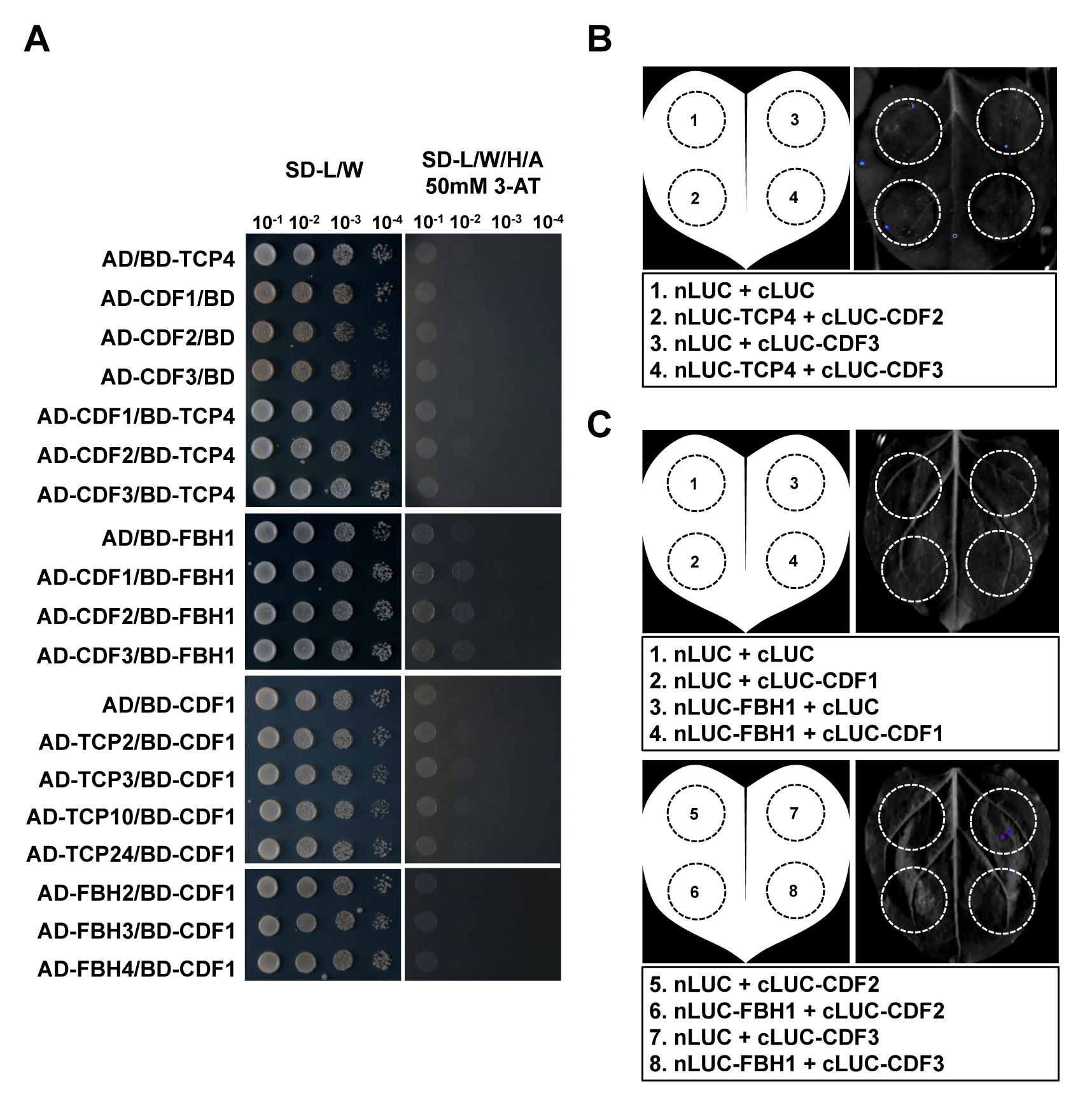

Supplement: S7 Fig — (A) Y2H assays to determine the interactions among TCPs-CDFs and FBHs-CDFs (n = 3). SD-L/W, synthetic dextrose medium lacking Leu and Trp; SD-L/W/H/A, synthetic dextrose medium lacking Leu, Trp, His and Ade; 3-AT, 3-amino-1,2,4-triazole; 10−1, 10−2, 10−3 and 10−4 denote the different dilution series. (B) LCI assays to detect the interaction between TCP4 and CDFs transcription factors (n = 5). (C) LCI assays to detect the interaction between FBH1 and CDFs transcription factors (n = 5). (TIF) [file pgen.1006833.s007.tif]

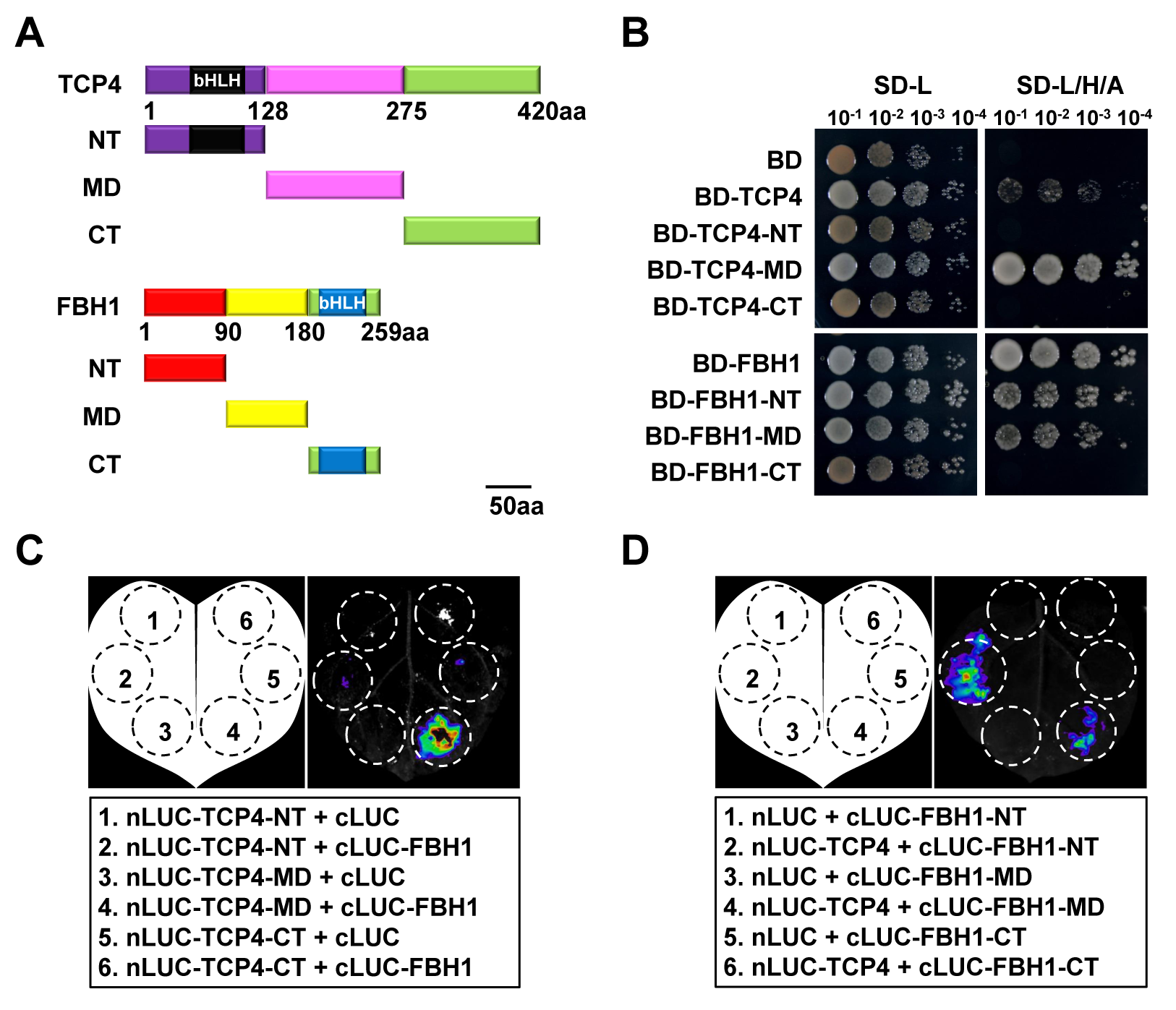

Supplement: S8 Fig — (A) Schemes display full length structures of TCP4 and FBH1 proteins as well as truncated versions. The locations of bHLH domains in TCP4 and FBH1 are marked by black and blue boxes, respectively. NT, amino terminal; MD, middle domain; CT, carboxyl terminal. Scale bar = 50 amino acids (aa). (B) Determination of the transcriptional activation domains of TCP4 and FBH1 in yeast. SD-L, synthetic dextrose medium lacking Leu; SD-L/H/A, synthetic dextrose medium lacking Leu, His and Ade; 10−1, 10−2, 10−3 and 10−4 denote the different dilution series. (C) LCI assay showing the interaction between truncated TCP4 versions and the full length FBH1. (D) LCI assay showing the interaction between full length TCP4 and the truncated FBH1 versions. The LUC signals in (C) and (D) were collected at 48 hpi (n = 5). (TIF) [file pgen.1006833.s008.tif]

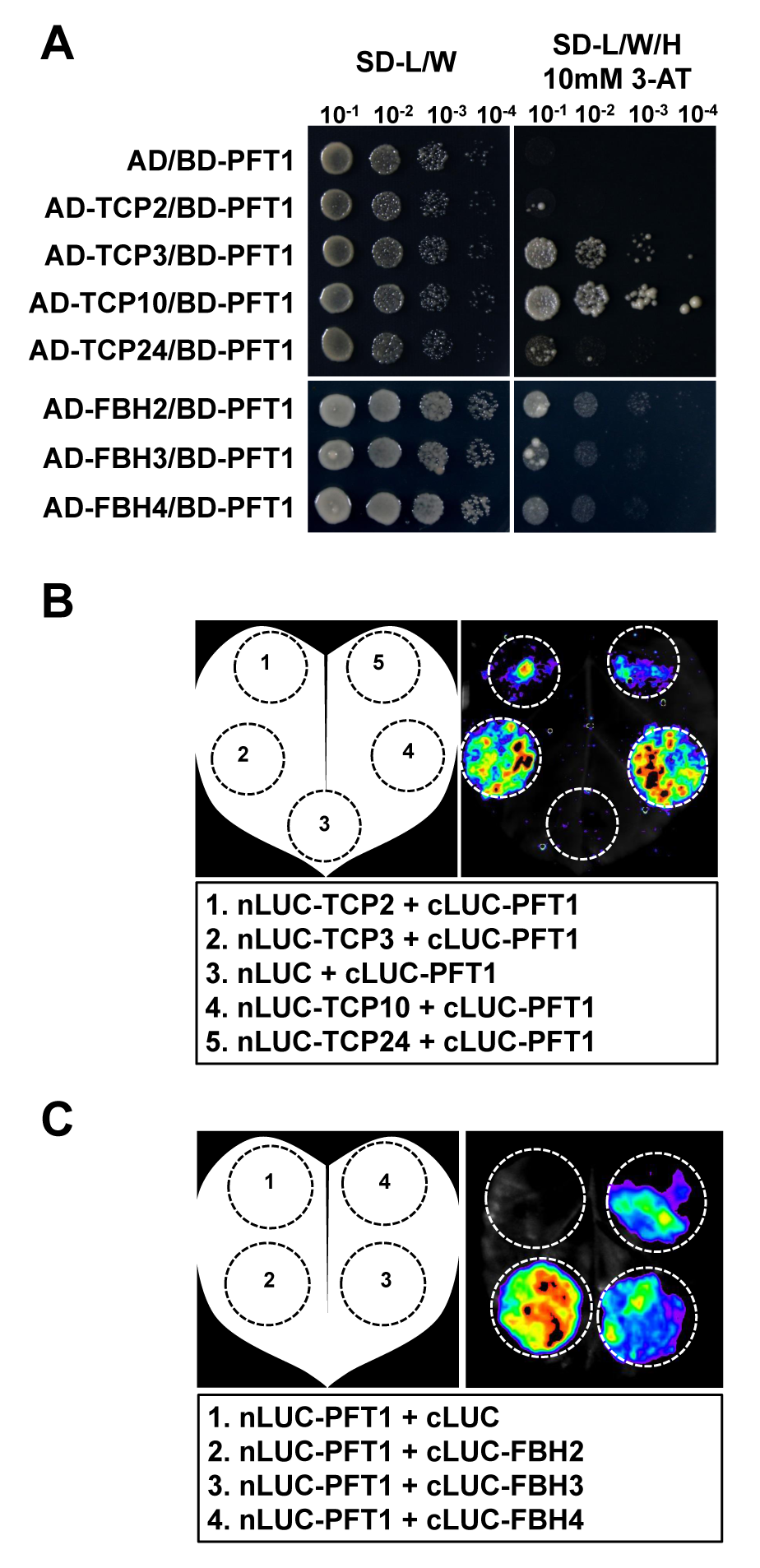

Supplement: S9 Fig — (A) Y2H assay showing the physical interaction between PFT1 and TCPs/FBHs (n = 3). (B) LCI assay showing the interaction between PFT1 and the miR319-regulated TCPs (n = 5). (C) LCI assay showing the interaction between PFT1 and FBHs (n = 5). (TIF) [file pgen.1006833.s009.tif]
