# Supplementary material for: MicroRNA319-regulated TCPs interact with FBHs and PFT1 to activate CO transcription and control flowering time in Arabidopsis
Source: PLoS Genet. 2017 May 30;13(5):e1006833. doi: 10.1371/journal.pgen.1006833 (PMC5469495; doi:10.1371/journal.pgen.1006833)
Supplement: S1 Table — (DOCX) [file pgen.1006833.s010.docx]

**Table S1** ***CO* promoter fragments used in yeast one-hybrid assay.**

| Fragment name | Sequence (5’-3’) |
| --- | --- |
| P8 | GAGATAAAACGAATCTAGCTTTACTTTAAATAAAATGCATATGAAAATAGTAAAAGGTGATACGAAAAAATAGTAACAATTTGCCTGCAACACCATGGCATTATCCGGACCACTTCCTCTTGAGAATCTCAGTATGGCAAGTGGCAAAACCTAAGCAACTTGTGAACGGGTCCCAACGAAGAAGTGCATAGGAGGAGATGTTTACACTTTACACTTTACACTTTACACTTTACACATAGGCCTTCCCAAAAGCTCAACTAGCTGCAAGAGGATCCAATAACATGTAAGAGCCACTAACGCTGTGCCACGTGTAGGCACTCAGGATTCGATCTCCCCTCTACTTATTCTCTCACACCAGATATAAGCTTTATTAGCCCCTTCTTTCAGATACCAGCTCCCACACCATCAAACTTACTACATCTGAGTTATT |
| P3 | GTATGTTAAGTTCCAATTACCAAGCAAAAAAAAACTTTTCAAAGTTTAAAGTTCAAAATGGGAAAGAGAAGTGCGGTGTAAGCAAATATGAAAGAGGAAGAGATGCGAAAAGTGTATCCTAGGACCAGCATTTTATACAAAAAAAAAACACTCACTTTTCAGCTCTTAAGGCATAGAGTGAAGGTAGCCATATGAATTTGGCCACTAGAGCGTCCGTCAAATCTCATTCTTTTTGGACCACATAATGGGTATCATACATTCACTGGACCCAAAAGCGTAACTGGAGCTAGTCCTCAAACCTAGAGAGTATCGTATCCTGTA |
| mP8 | GAGATAAAACGAATCTAGCTTTACTTTAAATAAAATGCATATGAAAATAGTAAAAGGTGATACGAAAAAATAGTAACAATTTGCCTGCAACACCATGGCATTATCCAAAAAACTTCCTCTTGAGAATCTCAGTATGGCAAGTGGCAAAACCTAAGCAACTTGTGAACAAAAAACAACGAAGAAGTGCATAGGAGGAGATGTTTACACTTTACACTTTACACTTTACACTTTACACATAGGCCTTCCCAAAAGCTCAACTAGCTGCAAGAGGATCCAATAACATGTAAGAGCCACTAACGCTGTGCCACGTGTAGGCACTCAGGATTCGATCTCCCCTCTACTTATTCTCTCACACCAGATATAAGCTTTATTAGCCCCTTCTTTCAGATACCAGCTCCCACACCATCAAACTTACTACATCTGAGTTATT |
| mP3 | GTATGTTAAGTTCCAATTACCAAGCAAAAAAAAACTTTTCAAAGTTTAAAGTTCAAAATGGGAAAGAGAAGTGCGGTGTAAGCAAATATGAAAGAGGAAGAGATGCGAAAAGTGTATCCTAAAAAAAGCATTTTATACAAAAAAAAAACACTCACTTTTCAGCTCTTAAGGCATAGAGTGAAGGTAGCCATATGAATTTGGCCACTAGAGCGTCCGTCAAATCTCATTCTTTTTAAAAAACATAATGGGTATCATACATTCACTAAAAAAAAAAGCGTAACTGGAGCTAGTCCTCAAACCTAGAGAGTATCGTATCCTGTA |

Note: The wild type TBM *cis*-elements and the mutated TBM sites are separately marked by blue and red color.
