# Supplementary material for: MicroRNA319-regulated TCPs interact with FBHs and PFT1 to activate CO transcription and control flowering time in Arabidopsis
Source: PLoS Genet. 2017 May 30;13(5):e1006833. doi: 10.1371/journal.pgen.1006833 (PMC5469495; doi:10.1371/journal.pgen.1006833)
Supplement: S2 Table — (DOCX) [file pgen.1006833.s011.docx]

**Table S2 Probes used in EMSA experiment.**

| Probe name | Sequence (5’-3’) |
| --- | --- |
| P8 Probe | GCCTGCAACACCATGGCATTATCCGGACCACTTCCTCTT |
| P3 Probe | CGTCCGTCAAATCTCATTCTTTTTGGACCACATAATGGG |
| P8 Competitor mut | GCCTGCAACACCATGGCATTATCCAAAAAACTTCCTCTT |
| P3 Competitor mut | CGTCCGTCAAATCTCATTCTTTTTAAAAAACATAATGGG |
