# Supplementary material for: MicroRNA319-regulated TCPs interact with FBHs and PFT1 to activate CO transcription and control flowering time in Arabidopsis
Source: PLoS Genet. 2017 May 30;13(5):e1006833. doi: 10.1371/journal.pgen.1006833 (PMC5469495; doi:10.1371/journal.pgen.1006833)
Supplement: S3 Table — (DOCX) [file pgen.1006833.s012.docx]

**Table S3. Primers used for qRT-PCR.**

| Primer name | Forward primer (5'-3') | Reverse primer (5'-3') |
| --- | --- | --- |
| miR319 | CGGTCTTGGACTGAAGGGAG | GTGCAGGGTCCGAGGT |
| *CO* | CAACAGCTTCACACCCAAGAACG | TTGCAGGGTCAGGTTGTTGCTC |
| *FT* | GCTACAACTGGAACAACCTTTGGC | TGAATTCCTGCAGTGGGACTTGG |
| *TCP4* | CCTTCAACGACGTCGTTTCAGCCAG | GTGAACCGGTGGAGGAAGGTGATG |
| *TCP2* | CGTCACCTACTACTACTAACTCCAAGC | CTGAAATGATTTTTAACCACAAGC |
| *TCP3* | TAGCTTCAACGCAACAGAGC | GGTTCTGTGTATTGCCTCGTG |
| *TCP10* | CCACGGAGAAGAAGCTACTCA | TCATCATGAATTTGAACCTCCA |
| *TCP24* | GCTCATGACAAGAATCTGAAGAAA | TGTTGCAGTGATAAACTTTTGAATTT |
| *U6* | GGGGACATCCGATAAAATTGG | GGACCATTTCTCGATTTATGC |
| *PFT1* | GAAAGCCCAATCAACAAAGTG | TTCACAGTAGCAGTTGGAACT |
| *ACT7* | TCCATGAAACAACTTACAACTCCATCA | CATCGTACTCACTCTTTGAAATCCACA |
| *NbACT1* | CCAAAGGCTAATCGTGAAAAG | GCTGTGGTAGTGGATGAGTAAC |
