# Supplementary material for: MicroRNA319-regulated TCPs interact with FBHs and PFT1 to activate CO transcription and control flowering time in Arabidopsis
Source: PLoS Genet. 2017 May 30;13(5):e1006833. doi: 10.1371/journal.pgen.1006833 (PMC5469495; doi:10.1371/journal.pgen.1006833)
Supplement: S4 Table — (DOCX) [file pgen.1006833.s013.docx]

**Table S4. Primers used for ChIP-qPCR assays.**

| Primer name | Forward primer (5'-3') | Reverse primer (5'-3') |
| --- | --- | --- |
| CO_pro_-P1 | TGAGAGAAGTTCCAAACCGCT | GTTGAGGCATCTTTGGCTTTTAG |
| CO_pro_-P2 | TCCAAAAATGCTTACTAAAGAACCTA | AAGTTCTTGTTATTTACTGGTTTTACG |
| CO_pro_-P3 | GTGTATCCTAGGACCAGCATT | GAGGACTAGCTCCAGTTACGC |
| CO_pro_-P4 | ATGAGCATAACACCAACAAGGC | CATGATCCATAGATGCAATTTAACC |
| CO_pro_-P5 | CTCTGTGATCTTAGAAACATGTCCTG | GTTGTCACATCTCTGTAATTTGGG |
| CO_pro_-P6 | AGTTTTAACTTGTGCGTCAGGT | TCATGGTAATGAGAATCATATCGG |
| CO_pro_-P7 | CATTCACAAATAATGGAACAACAACT | GATTCGTTTTATCTCTTTGGCTTTAT |
| CO_pro_-P8 | GCCTGCAACACCATGGCATTA | GGATCCTCTTGCAGCTAGTTG |
