# Supplementary material for: MicroRNA319-regulated TCPs interact with FBHs and PFT1 to activate CO transcription and control flowering time in Arabidopsis
Source: PLoS Genet. 2017 May 30;13(5):e1006833. doi: 10.1371/journal.pgen.1006833 (PMC5469495; doi:10.1371/journal.pgen.1006833)
Supplement: S5 Table — (DOCX) [file pgen.1006833.s014.docx]

**Table S5. Primers used for site-directed mutagenesis.**

| Primer name | Forward primer (5'-3') | Reverse primer (5'-3') |
| --- | --- | --- |
| mP8 | GGCATTATCCAAAAAACTTCCTCTTGAGAATCTCAGTATGG | CAAGAGGAAGTTTTTTGGATAATGCCATGGTGTTGCAGGCA |
|  | ACTTGTGAACAAAAAACAACGAAGAAGTGCATAGGAGGAGA | TTCTTCGTTGTTTTTTGTTCACAAGTTGCTTAGGTTTTGC |
| mP3 | GTGTATCCTAAAAAAAGCATTTTATACAAAAAAAAAACA | GTATAAAATGCTTTTTTTAGGATACACTTTTCGCATCTC |
|  | TAATGGGTATCATACATTCACTAAAAAAAAAAGCG | GAATGTATGATACCCATTATGTTTTTTAAAAAGAATGA |
| rTCP2 | GACCtTgCAaagtAAcTCAACAAATCAGCATCAGTC | TTGAgTTacttTGcAaGGTCCCCCTGTTGAAACCAC |
| rTCP3 | GGTCCCtTgCAaagtATcAACACACCTATGATTCGTGC | GTGTTgATacttTGcAaGGGACCCCTCTGTGATAAAAG |
| rTCP4 | GGTCCCtTgCAaagtAGcTACAGTCCCATGATCCGTGC | CTGTAgCTacttTGcAaGGGACCCCTCTGAGAATAAA |
| rTCP10 | GTACCtTgCAaagtAGcTTATTCCCTCATTCGTTTCG | GAATAAgCTacttTGcAaGGTACCCCTTTGTTGTTGTT |
| rTCP24 | GGACCtTgCAaagtAAcTCACAATCTCTCTTTCTGAA | TGTGAgTTacttTGcAaGGTCCCCCTACTACTGTAAC |
