# Supplementary material for: MicroRNA319-regulated TCPs interact with FBHs and PFT1 to activate CO transcription and control flowering time in Arabidopsis
Source: PLoS Genet. 2017 May 30;13(5):e1006833. doi: 10.1371/journal.pgen.1006833 (PMC5469495; doi:10.1371/journal.pgen.1006833)
Supplement: S6 Table — (DOCX) [file pgen.1006833.s015.docx]

**Table S6. Constructs used in this study.**

| Construct name | Vector | Description |
| --- | --- | --- |
| TCP4_pro_:GUS | pMDC162 | Spatial expression pattern assay of TCP4 |
| CO_pro_:LUC | pGWB35 | Transcriptional activity assay in *Nicotiana* |
| 35S:rTCP2 | pGWB17 | Transcriptional activity assay in *Nicotiana* |
| 35S:rTCP3 | pGWB17 | Transcriptional activity assay in *Nicotiana* |
| 35S:rTCP4 | pGWB17 | Transcriptional activity assay in *Nicotiana* |
| 35S:rTCP10 | pGWB17 | Transcriptional activity assay in *Nicotiana* |
| 35S:rTCP24 | pGWB17 | Transcriptional activity assay in *Nicotiana* |
| 35S:FBH1 | pGWB17 | Transcriptional activity assay in *Nicotiana* |
| 35S:PFT1 | pGWB17 | Transcriptional activity assay in *Nicotiana* |
| pERGW-rTCP4 | pERGW | Transcriptional activity assay in *Nicotiana* |
| nLUC-TCP2 | p1300-35S-nLUC | LCI |
| nLUC-TCP3 | p1300-35S-nLUC | LCI |
| nLUC-TCP4 | p1300-35S-nLUC | LCI |
| nLUC-TCP10 | p1300-35S-nLUC | LCI |
| nLUC-TCP24 | p1300-35S-nLUC | LCI |
| nLUC-TCP4-NT | p1300-35S-nLUC | LCI |
| nLUC-TCP4-MD | p1300-35S-nLUC | LCI |
| nLUC-TCP4-CT | p1300-35S-nLUC | LCI |
| nLUC-PFT1 | p1300-35S-nLUC | LCI |
| cLUC-FBH1 | p1300-35S-nLUC | LCI |
| cLUC-FBH1-NT | p1300-35S-nLUC | LCI |
| cLUC-FBH1-MD | p1300-35S-nLUC | LCI |
| cLUC-FBH1-CT | p1300-35S-nLUC | LCI |
| cLUC-CDF1 | p1300-35S-cLUC | LCI |
| cLUC-CDF2 | p1300-35S-cLUC | LCI |
| cLUC-CDF3 | p1300-35S-cLUC | LCI |
| cLUC-FBH1 | p1300-35S-cLUC | LCI |
| cLUC-FBH2 | p1300-35S-cLUC | LCI |
| cLUC-FBH3 | p1300-35S-cLUC | LCI |
| cLUC-FBH4 | p1300-35S-cLUC | LCI |
| cLUC-PFT1 | p1300-35S-cLUC | LCI |
| TCP4-GFP | pGWB5 | Co-IP |
| PFT1-GFP | pGWB5 | Co-IP |
| FBH1-GFP | pGWB5 | Co-IP |
| TCP4-Myc | pGWB17 | Co-IP |
| FBH1-Myc | pGWB17 | Co-IP |
| TCP4-CFP | HBT-CFP | FLIM-FRET |
| PFT1-CFP | HBT-CFP | FLIM-FRET |
| FBH1-YFP | HBT-YFP | FLIM-FRET |
| CDF1-YFP | HBT-YFP | FLIM-FRET |
| YFP | HBT-YFP | FLIM-FRET |
